# Sae2 Function at DNA Double-Strand Breaks Is Bypassed by Dampening Tel1 or Rad53 Activity

**DOI:** 10.1371/journal.pgen.1005685

**Published:** 2015-11-19

**Authors:** Elisa Gobbini, Matteo Villa, Marco Gnugnoli, Luca Menin, Michela Clerici, Maria Pia Longhese

**Affiliations:** Dipartimento di Biotecnologie e Bioscienze, Università di Milano-Bicocca, Milano, Italy; Duke University, UNITED STATES

## Abstract

The MRX complex together with Sae2 initiates resection of DNA double-strand breaks (DSBs) to generate single-stranded DNA (ssDNA) that triggers homologous recombination. The absence of Sae2 not only impairs DSB resection, but also causes prolonged MRX binding at the DSBs that leads to persistent Tel1- and Rad53-dependent DNA damage checkpoint activation and cell cycle arrest. Whether this enhanced checkpoint signaling contributes to the DNA damage sensitivity and/or the resection defect of *sae2*Δ cells is not known. By performing a genetic screen, we identify *rad53* and *tel1* mutant alleles that suppress both the DNA damage hypersensitivity and the resection defect of *sae2*Δ cells through an Sgs1-Dna2-dependent mechanism. These suppression events do not involve escaping the checkpoint-mediated cell cycle arrest. Rather, defective Rad53 or Tel1 signaling bypasses Sae2 function at DSBs by decreasing the amount of Rad9 bound at DSBs. As a consequence, reduced Rad9 association to DNA ends relieves inhibition of Sgs1-Dna2 activity, which can then compensate for the lack of Sae2 in DSB resection and DNA damage resistance. We propose that persistent Tel1 and Rad53 checkpoint signaling in cells lacking Sae2 increases the association of Rad9 at DSBs, which in turn inhibits DSB resection by limiting the activity of the Sgs1-Dna2 resection machinery.

## Author Summary

Genome instability is one of the most pervasive characteristics of cancer cells and can be due to DNA repair defects and failure to arrest the cell cycle. Among the many types of DNA damage, the DNA double strand break (DSB) is one of the most severe, because it can cause mutations and chromosomal rearrangements. Generation of DSBs triggers a highly conserved mechanism, known as DNA damage checkpoint, which arrests the cell cycle until DSBs are repaired. DSBs can be repaired by homologous recombination, which requires the DSB ends to be nucleolytically processed (resected) to generate single-stranded DNA. In *Saccharomyces cerevisiae*, DSB resection is initiated by the MRX complex together with Sae2, whereas more extensive resection is catalyzed by both Exo1 and Dna2-Sgs1. The absence of Sae2 not only impairs DSB resection, but also leads to the hyperactivation of the checkpoint proteins Tel1/ATM and Rad53, leading to persistent cell cycle arrest. In this manuscript we show that persistent Tel1 and Rad53 signaling activities in *sae2*Δ cells cause DNA damage hypersensitivity and defective DSB resection by increasing the amount of Rad9 bound at the DSBs, which in turn inhibits the Sgs1-Dna2 resection machinery. As ATM inhibition has been proposed as a strategy for cancer treatment, the finding that defective Tel1 signaling activity restores DNA damage resistance in *sae2*Δ cells might have implications in cancer therapies that use ATM inhibitors for synthetic lethal approaches that are devised to kill tumor cells with defective DSB repair.

## Introduction

Programmed DNA double-strand breaks (DSBs) are formed during meiotic recombination and rearrangement of the immunoglobulin genes in lymphocytes. Furthermore, potentially harmful DSBs can arise by exposure to environmental factors, such as ionizing radiations and radiomimetic chemicals, or by failures in DNA replication. DSB generation elicits a checkpoint response that depends on the mammalian protein kinases ATM and ATR, whose functional orthologs in *Saccharomyces cerevisiae* are Tel1 and Mec1, respectively [1]. Tel1/ATM is recruited to DSBs by the MRX (Mre11-Rad50-Xrs2)/MRN (Mre11-Rad50-Nbs1) complex, whereas Mec1/ATR recognizes single-stranded DNA (ssDNA) covered by Replication Protein A (RPA) [2]. Once activated, Tel1/ATM and Mec1/ATR propagate their checkpoint signals by phosphorylating the downstream checkpoint kinases Rad53 (Chk2 in mammals) and Chk1, to couple cell cycle progression with DNA repair [2].

Repair of DSBs can occur by either non-homologous end joining (NHEJ) or homologous recombination (HR). Whereas NHEJ directly joins the DNA ends, HR uses the sister chromatid or the homologous chromosome to repair DSBs. HR requires that the 5’ ends of a DSB are nucleolytically processed (resected) to generate 3’-ended ssDNA that can invade an undamaged homologous DNA template [3,4]. In *Saccharomyces cerevisiae*, recent characterization of core resection proteins has revealed that DSB resection is initiated by the MRX complex, which catalyzes an endonucleolytic cleavage near a DSB [4], with the Sae2 protein (CtIP in mammals) promoting MRX endonucleolytic activity [5]. This MRX-Sae2-mediated DNA clipping generates 5’ DNA ends that are optimal substrates for the nucleases Exo1 and Dna2, the latter working in concert with the helicase Sgs1 [6–9]. In addition, the MRX complex recruits Exo1, Sgs1 and Dna2 to DSBs independently of the Mre11 nuclease activity [10]. DSB resection is also negatively regulated by Ku and Rad9, which inhibit the access to DSBs of Exo1 and Sgs1-Dna2, respectively [11–14].

The MRX-Sae2-mediated endonucleolytic cleavage is particularly important to initiate resection at DNA ends that are not easily accessible to Exo1 and Dna2-Sgs1. For instance, both *sae2*Δ and *mre11* nuclease defective mutants are completely unable to resect meiotic DSBs, where the Spo11 topoisomerase-like protein remains covalently attached to the 5’-terminated strands [15,16]. Furthermore, the same mutants exhibit a marked sensitivity to camptothecin (CPT), which extends the half-life of DNA-topoisomerase I cleavable complexes [17,18], and to methyl methanesulfonate (MMS), which can generate chemically complex DNA termini. The lack of Rad9 or Ku suppresses both the hypersensitivity to DSB-inducing agents and the resection defect of *sae2*Δ cells [10–14]. These suppression events require Dna2-Sgs1 and Exo1, respectively, indicating that Rad9 increases the requirement for MRX-Sae2 activity in DSB resection by inhibiting Sgs1-Dna2 [13,14], while Ku mainly limits the action of Exo1 [10–12]. By contrast, elimination of either Rad9 or Ku does not bypass Sae2/MRX function in resecting meiotic DSBs [11,13], likely because Sgs1-Dna2 and Exo1 cannot substitute for the Sae2/MRX-mediated endonucleolytic cleavage when this event is absolutely required to generate accessible 5’-terminated DNA strands.

Sae2 plays an important role also in modulating the checkpoint response. Checkpoint activation in response to DSBs depends primarily on Mec1, with Tel1 playing a minor role [19]. On the other hand, impaired Mre11 endonuclease activity caused by the lack of Sae2 leads to increased MRX persistence at the DSB ends. The enhanced MRX signaling in turn causes unscheduled Tel1-dependent checkpoint activation that is associated to prolonged Rad53 phosphorylation [20–22]. Mutant *mre11* alleles that reduce MRX binding to DSBs restore DNA damage resistance in *sae2*Δ cells and reduce their persistent checkpoint activation without restoring efficient DSB resection [23,24], suggesting that enhanced MRX association to DSBs contributes to the DNA damage hypersensitivity caused by the lack of Sae2. Persistently bound MRX might increase the sensitivity to DNA damaging agents of *sae2*Δ cells by hyperactivating the DNA damage checkpoint. If this were the case, then the DNA damage hypersensitivity of *sae2*Δ cells should be restored by the lack of Tel1 or of its downstream effector Rad53, as they are responsible for the *sae2*Δ enhanced checkpoint signaling [20,22]. However, while Rad53 inactivation has never been tested, *TEL1* deletion not only fails to restore DNA damage resistance in *sae2*Δ cells, but it exacerbates their sensitivity to DNA damaging agents [23,24]. Therefore, other studies are required to understand whether the Tel1- and Rad53-mediated checkpoint signaling has any role in determining the DNA damage sensitivity of *sae2*Δ cells.

By performing a genetic screen, we identified *rad53* and *tel1* mutant alleles that suppress both the hypersensitivity to DNA damaging agents and the resection defect of *sae2*Δ cells by reducing the amount of Rad9 at DSBs. Decreased Rad9 binding at DNA ends bypasses Sae2 function in DNA damage resistance and resection by relieving the inhibition of the Sgs1-Dna2 resection machinery. Altogether our data suggest that the primary cause of the resection defect of *sae2*Δ cells is Rad9 association to DSBs, which is promoted by persistent Tel1 and Rad53 signaling activities in these cells.

## Results

### The Rad53-H88Y and Tel1-N2021D variants suppress the DNA damage hypersensitivity of *sae2*Δ cells

**Fig 1:**
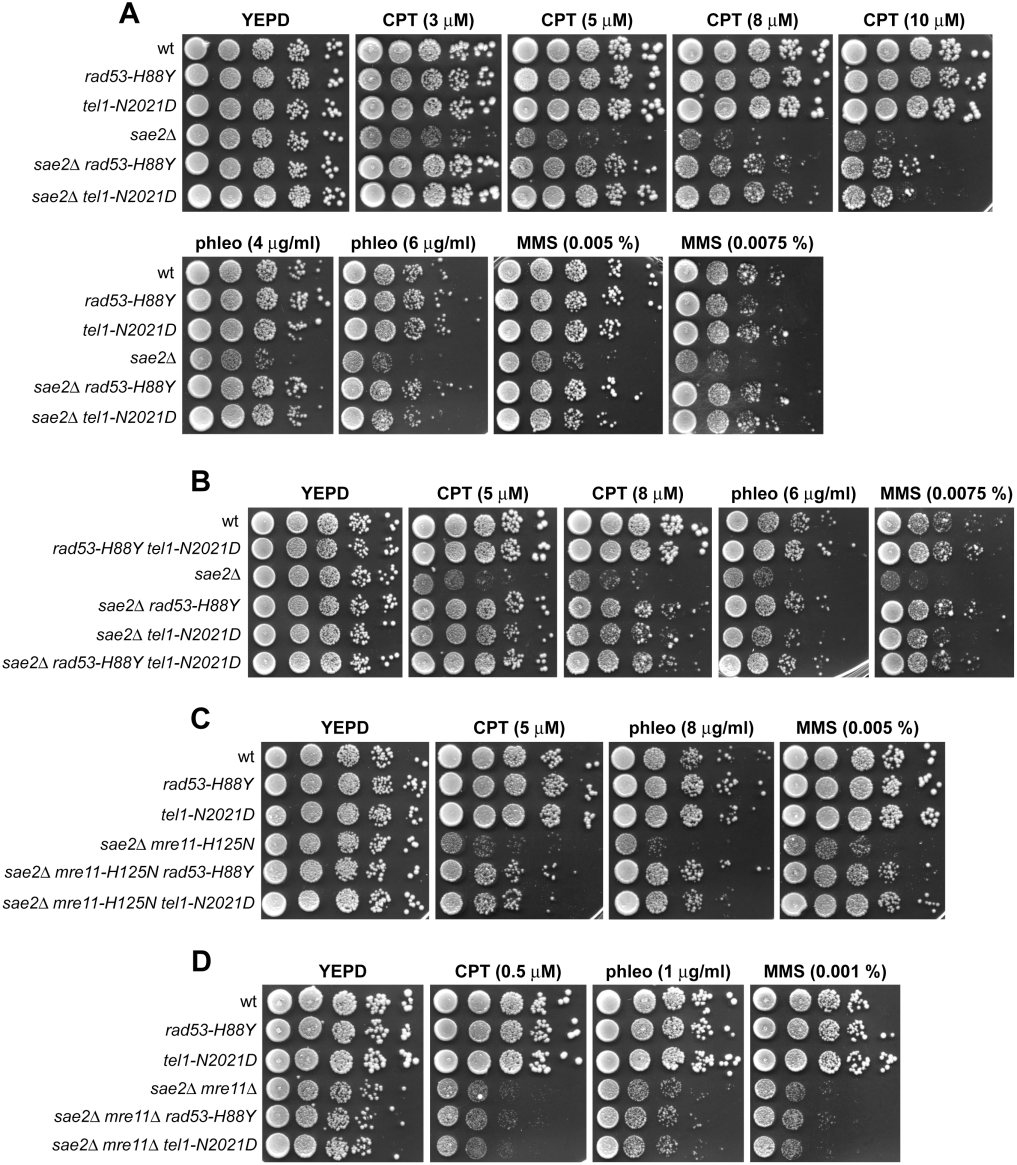
Rad53-H88Y and Tel1-N2021D suppress the hypersensitivity to genotoxic agents of *sae2*Δ cells. (A-D) Exponentially growing cells were serially diluted (1:10) and each dilution was spotted out onto YEPD plates with or without CPT, phleomycin or MMS.

We have previously described our search for extragenic mutations that suppress the CPT hypersensitivity of *sae2*Δ cells [13]. This genetic screen identified 15 single-gene suppressor mutants belonging to 11 distinct allelism groups. Analysis of genomic DNA by next-generation Illumina sequencing of 5 non allelic suppressor mutants revealed that the DNA damage resistance was due to single base pair substitutions in the genes encoding Sgs1, Top1, or the multi-drug resistance proteins Pdr3, Pdr10 and Sap185 [13]. Subsequent genome sequencing and genetic analysis of 2 more non allelic suppressor mutants allowed to link suppression to either the *rad53-H88Y* mutant allele, causing the replacement of Rad53 amino acid residue His88 by Tyr, or the *tel1-N2021D* allele, resulting in the replacement of Tel1 amino acid residue Asn2021 by Asp. Both *rad53-H88Y* and *tel1-N2021D* alleles restored resistance of *sae2*Δ cells not only to CPT, but also to phleomycin (phleo) and MMS (Fig 1A). While both *rad53-H88Y* and *tel1-N2021D* fully rescued the hypersensitivity of *sae2*Δ cells to phleomycin and MMS, the CPT hypersensitivity of *sae2*Δ cells was only partially suppressed by the same alleles (Fig 1A), suggesting that they did not bypass all Sae2 functions.

Both *rad53-H88Y* and *tel1-N2021D* suppressor alleles were recessive, as the sensitivity to genotoxic agents of *sae2*Δ/*sae2*Δ *RAD53*/*rad53-H88Y* and *sae2*Δ/*sae2*Δ *TEL1*/*tel1-N2021D* diploid cells was similar to that of *sae2*Δ/*sae2*Δ *RAD53/RAD53 TEL1/TEL1* diploid cells (S1 Fig), suggesting that *rad53-H88Y* and *tel1-N2021D* alleles encode hypomorphic variants. Furthermore, both variants suppressed the hypersensitivity to DNA damaging agents of *sae2*Δ cells by altering the same mechanism, as *sae2*Δ *rad53-H88Y tel1-N2021D* triple mutant cells survived in the presence of DNA damaging agents to the same extent as *sae2*Δ *rad53-H88Y* and *sae2*Δ *tel1-N2021D* double mutant cells (Fig 1B).

The MRX complex not only provides the nuclease activity for initiation of DSB resection, but also it promotes the binding of Exo1, Sgs1 and Dna2 at the DSB ends [10]. These MRX multiple roles explain the severe DNA damage hypersensitivity and resection defect of cells lacking any of the MRX subunits compared to cells lacking either Sae2 or the Mre11 nuclease activity. As Sae2 has been proposed to activate Mre11 nuclease activity [5], we asked whether the suppression of *sae2*Δ DNA damage hypersensitivity by Rad53-H88Y and Tel1-N2021D requires Mre11 nuclease activity. Both *rad53-H88Y* and *tel1-N2021D* alleles suppressed the hypersensitivity to DNA damaging agents of *sae2*Δ cells carrying the nuclease defective *mre11-H125N* allele (Fig 1C). By contrast, *sae2*Δ *mre11*Δ *rad53-H88Y* and *sae2*Δ *mre11*Δ *tel1-N2021D* triple mutant cells were as sensitive to genotoxic agents as *sae2*Δ *mre11*Δ double mutant cells (Fig 1D), indicating that neither the *rad53-H88Y* nor the *tel1-N2021D* allele can suppress the hypersensitivity to DNA damaging agents of *sae2*Δ *mre11*Δ cells. Altogether, these findings indicate that both Rad53-H88Y and Tel1-N2021D require the physical presence of the MRX complex, but not its nuclease activity, to bypass Sae2 function in cell survival to genotoxic agents.

### The Rad53-H88Y variant is defective in the interaction with Rad9 and bypasses the adaptation defect of *sae2*Δ cells by impairing checkpoint activation

A single unrepairable DSB induces a DNA damage checkpoint that depends primarily on Mec1, with Tel1 playing a minor role [19]. This checkpoint response can be eventually turned off, allowing cells to resume cell cycle progression through a process that is called adaptation [25–27]. In the absence of Sae2, cells display heightened checkpoint activation that prevents cells from adapting to an unrepaired DSB [20,22]. This persistent checkpoint activation is due to increased MRX amount/persistence at the DSB that in turn causes enhanced and prolonged Tel1 activation that is associated with persistent Rad53 phosphorylation [20–22,28].

**Fig 2:**
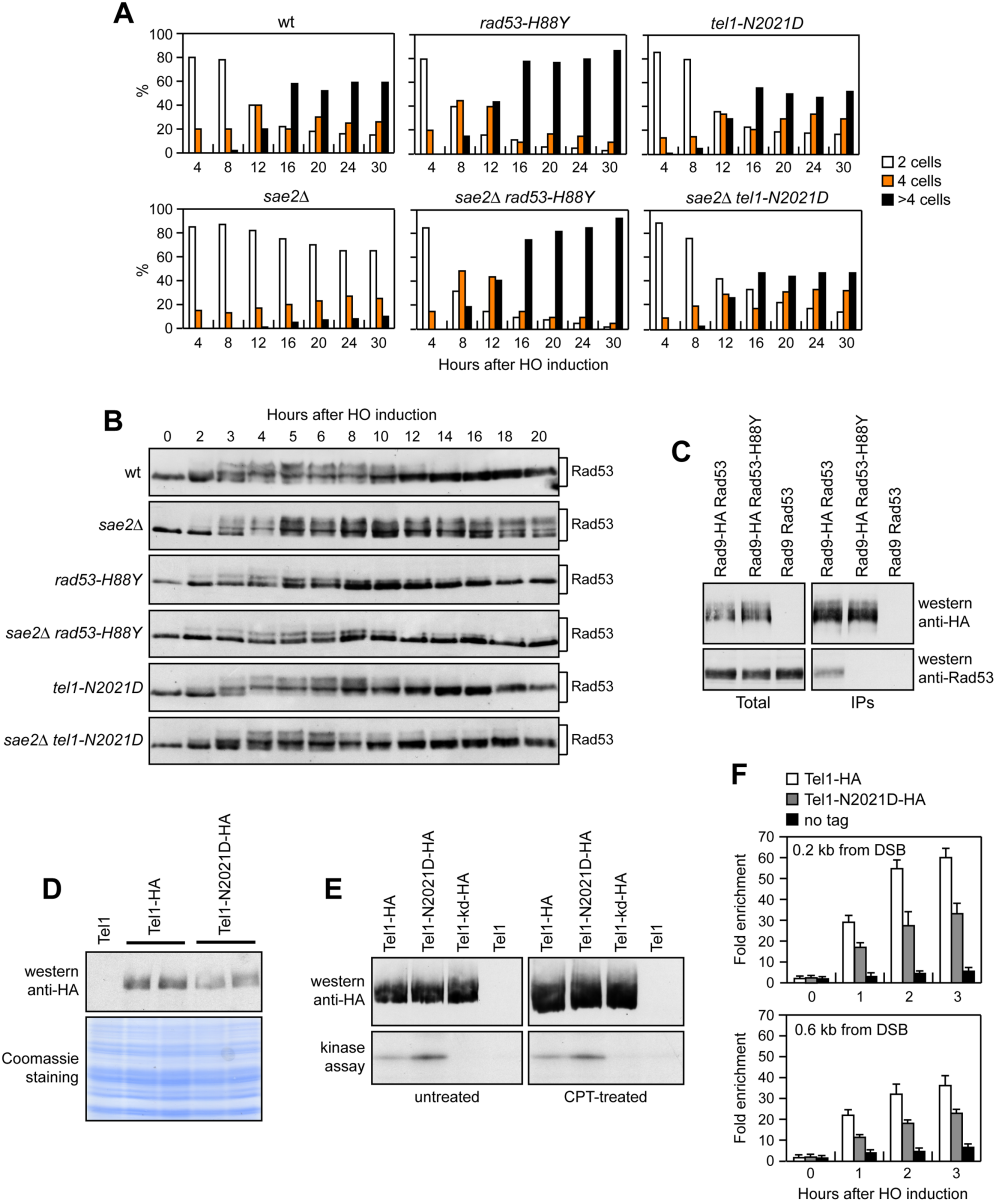
Rad53-H88Y and Tel1-N2021D suppress the checkpoint shut off defect of *sae2*Δ cells. (A) G1-arrested cell cultures of JKM139 derivative strains were plated on galactose-containing plates (time zero). At the indicated time points, 200 cells for each strain were analyzed to determine the frequency of large budded cells (2 cells) and of cells forming microcolonies of 4 or more than 4 cells. (B) Exponentially growing YEPR cultures of the strains in (A) were transferred to YEPRG (time zero), followed by western blot analysis with anti-Rad53 antibodies. (C) Protein extracts were analyzed by western blot with anti-HA or anti-Rad53 antibodies either directly (Total) or after Rad9-HA immunoprecipitation (IPs) with anti-HA antibodies. (D) Protein extracts from exponentially growing cells were analyzed by western blotting with anti-HA antibodies. The same amounts of protein extracts were separated by SDS-PAGE and stained with Coomassie as loading control. (E) Kinase assay was performed on equal amounts of anti-HA immunoprecipitates of protein extracts from cells either exponentially growing in YEPD or after treatment with 50μM CPT for 1 hour. All the immunoprecipitates were also subjected to western blot analysis using anti-HA antibodies. (F) Relative fold enrichment of Tel1-HA and Tel1-N2021D-HA compared to untagged Tel1 (no tag) at the indicated distance from the HO cleavage site was evaluated after ChIP with anti-HA antibodies and qPCR analysis. In all diagrams, the ChIP signals were normalized for each time point to the amount of the corresponding immunoprecipitated protein and input signal. The mean values are represented with error bars denoting s.d. (n = 3).

If the *rad53-H88Y* mutation impaired Rad53 activity, then it is expected to suppress the adaptation defect of *sae2*Δ cells by lowering checkpoint activation. We addressed this point by using JKM139 derivative strains, where a single DSB at the *MAT* locus can be generated by expression of the HO endonuclease gene under the control of a galactose-dependent promoter. This DSB cannot be repaired by HR because of the deletion of the homologous donor loci *HML* and *HMR* [27]. We measured checkpoint activation by monitoring the ability of cells to arrest the cell cycle and to phosphorylate Rad53 after HO induction. Both *rad53-H88Y* and *sae2*Δ *rad53-H88Y* cells formed microcolonies of more than 2 cells with higher efficiency than either wild type or *sae2*Δ cells (Fig 2A). Furthermore, the Rad53-H88Y variant was poorly phosphorylated after HO induction both in the presence and in the absence of Sae2 (Fig 2B). Thus, the *rad53-H88Y* mutation suppresses the adaptation defect of *sae2*Δ cells by impairing Rad53 activation.

DNA damage-dependent activation of Rad53 requires its phospho-dependent interaction with Rad9, which acts as a scaffold to allow Rad53 intermolecular authophosphorylation and activation [29–31]. Interestingly, the His88 residue, which is replaced by Tyr in the Rad53-H88Y variant, is localized in the forkhead-associated domain 1 of the protein and has been implicated in mediating Rad9-Rad53 interaction [32]. Thus, we asked whether the Rad53-H88Y variant was defective in the interaction with Rad9. When HA-tagged Rad9 was immunoprecipitated with anti-HA antibodies from wild type and *rad53-H88Y* cells grown for 4 hours in the presence of galactose to induce HO, wild type Rad53 could be detected in Rad9-HA immunoprecipitates, whereas Rad53-H88Y did not (Fig 2C). This defective interaction of Rad53-H88Y with Rad9 could explain the impaired checkpoint activation in *sae2*Δ *rad53-H88Y* double mutant cells.

### The Tel1-N2021D variant binds poorly to DSBs and bypasses the adaptation defect of *sae2*Δ cells by reducing persistent Rad53 activation

Tel1 signaling activity is responsible for the prolonged Rad53 activation that prevents *sae2*Δ cells to adapt to the checkpoint triggered by an unrepairable DSB [20,22]. Although telomere length in *tel1-N2021D* mutant cells was unaffected both in the presence and in the absence of Sae2 (S2 Fig), the recessivity of *tel1-N2021D* suppressor effect on *sae2*Δ DNA damage hypersensitivity suggests that the N2021D substitution impairs Tel1 function. If this were the case, Tel1-N2021D might suppress the adaptation defect of *sae2*Δ cells by reducing the DSB-induced persistent Rad53 phosphorylation. When G1-arrested cell cultures were spotted on galactose-containing plates to induce HO, wild type, *sae2*Δ, *tel1-N2021D* and *sae2*Δ *tel1-N2021D* cells accumulated large budded cells within 4 hours (Fig 2A). This cell cycle arrest is due to checkpoint activation. In fact, when the same cells exponentially growing in raffinose were transferred to galactose, Rad53 phosphorylation was detectable about 2–3 hours after galactose addition (Fig 2B). However, while *sae2*Δ cells remained arrested as large budded cells for at least 30 hours (Fig 2A) and showed persistent Rad53 phosphorylation (Fig 2B), wild type, *tel1-N2021D* and *sae2*Δ *tel1-N2021D* cells formed microcolonies with more than 2 cells (Fig 2A) and decreased the amounts of phosphorylated Rad53 (Fig 2B) with similar kinetics 10–12 hours after HO induction. Therefore, the Tel1-N2021D variant impairs Tel1 signaling activity, as it rescues the *sae2*Δ adaptation defect by reducing the persistent Rad53 phosphorylation.

The N2021D substitution resides in the Tel1 FAT domain, a helical solenoid that encircles the kinase domain of all the phosphoinositide 3-kinase (PI3K)-related kinases (PIKKs) [33,34], suggesting that this amino acid change might reduce Tel1 kinase activity. Western blot analysis revealed that the amount of Tel1-N2021D was slightly lower than that of wild type Tel1 (Fig 2D). We then immunoprecipitated equivalent amounts of Tel1-HA and Tel1-N2021D-HA variants from both untreated and CPT-treated cells (Fig 2E, top), and we measured their kinase activity in vitro using the known artificial substrate of the PIKKs family PHAS-I (Phosphorylated Heat and Acid Stable protein) [35]. Both Tel1-HA and Tel1-N2021D-HA were capable to phosphorylate PHAS-I, with the amount of phosphorylated substrate being slighly higher in Tel1-N2021D-HA than in Tel1-HA immunoprecipitates (Fig 2E, bottom). This PHAS-I phosphorylation was dependent on Tel1 kinase activity, as it was not detectable when the immunoprecipitates were prepared from strains expressing either kinase dead Tel1-kd-HA or untagged Tel1 (Fig 2E, bottom). Thus, the *tel1-N2021D* mutation does not affect Tel1 kinase activity.

Interestingly, the FAT domain is in close proximity to the FATC domain, which was shown to be important for Tel1 recruitment to DNA ends [36], suggesting that the Tel1-N2021D variant might be defective in recruitment/association to DSBs. Strikingly, when we analyzed Tel1 and Tel1-N2021D binding at the HO-induced DSB by chromatin immunoprecipitation (ChIP) and quantitative real time PCR (qPCR), the amount of Tel1-N2021D bound at the DSB turned out to be lower than that of wild type Tel1 (Fig 2F). This decreased Tel1-N2021D association was not due to lower Tel1-N2021D levels, as the ChIP signals were normalized for each time point to the amount of immunoprecipitated protein. Thus, the inability of *sae2*Δ *tel1-N2021D* cells to sustain persistent Rad53 phosphorylation after DSB generation can be explained by a decreased association of Tel1-N2021D to DSBs.

### Checkpoint-mediated cell cycle arrest is not responsible for the DNA damage hypersensitivity of *sae2*Δ cells

**Fig 3:**
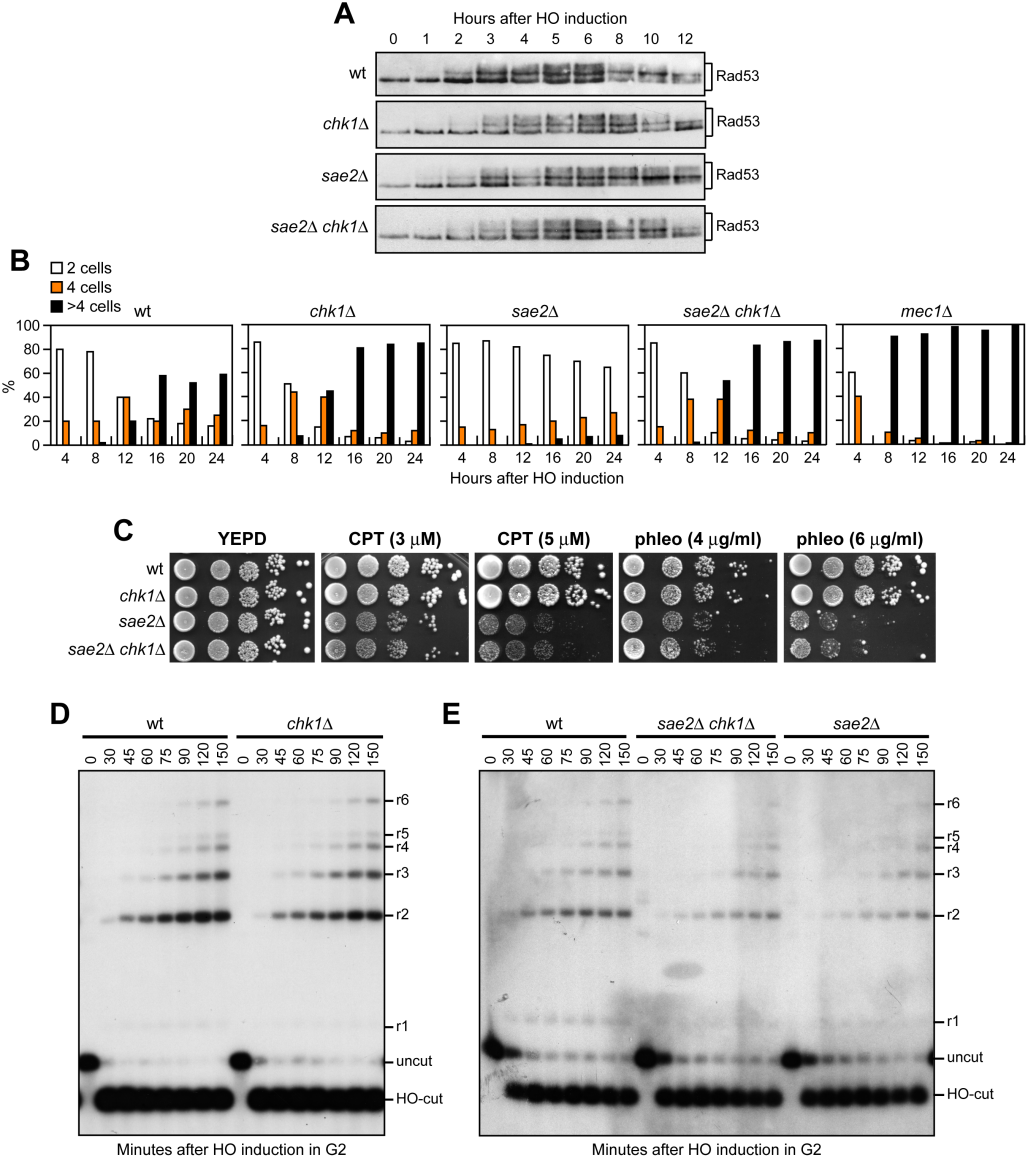
The lack of Chk1 does not suppress the hypersensitivity to DNA damaging agents of *sae2*Δ cells. (A) Exponentially growing YEPR cultures of JKM139 derivative strains were transferred to YEPRG (time zero), followed by western blot analysis with anti-Rad53 antibodies. (B) YEPR G1-arrested cell cultures of JKM139 derivative strains were plated on galactose-containing plates (time zero). At the indicated time points, 200 cells for each strain were analyzed to determine the frequency of large budded cells (2 cells) and of cells forming microcolonies of 4 or more than 4 cells. (C) Exponentially growing cells were serially diluted (1:10) and each dilution was spotted out onto YEPD plates with or without CPT and phleomycin. (D, E) DSB resection. YEPR exponentially growing cultures of JKM139 derivative cells were arrested in G2 with nocodazole and transferred to YEPRG in the presence of nocodazole at time zero. Gel blots of SspI-digested genomic DNA separated on alkaline agarose gel were hybridized with a single-stranded RNA probe that anneals to the unresected strand on one side of the break. 5’-3’ resection progressively eliminates SspI sites, producing larger SspI fragments (r1 through r6) detected by the probe.

As both Rad53-H88Y and Tel1-N2021D reduce checkpoint signaling in *sae2*Δ cells, we asked whether the increased DNA damage resistance of *sae2*Δ *rad53-H88Y* and *sae2*Δ *tel1-N2021D* cells was due to the elimination of the checkpoint-mediated cell cycle arrest. This hypothesis could not be tested by deleting the *MEC1*, *DDC1*, *RAD24*, *MEC3* or *RAD9* checkpoint genes, because they also regulate DSB resection [37–39]. On the other hand, an HO-induced DSB activates also the Chk1 checkpoint kinase [40], which contributes to arrest the cell cycle in response to DSBs by controlling a pathway that is independent of Rad53 [41]. Importantly, *chk1*Δ cells do not display DNA damage hypersensitivity and are not defective in resection of uncapped telomeres [38,41]. We therefore asked whether *CHK1* deletion restores DNA damage resistance in *sae2*Δ cells. Consistent with the finding that Chk1 contributes to arrest the cell cycle after DNA damage independently of Rad53 [41], Rad53 was phosphorylated with wild type kinetics after HO induction in both *chk1*Δ and *sae2*Δ *chk1*Δ cells (Fig 3A). Furthermore, *CHK1* deletion suppresses the adaptation defect of *sae2*Δ cells. In fact, both *chk1*Δ and *sae2*Δ *chk1*Δ cells spotted on galactose-containing plates formed microcolonies of more than 2 cells with higher efficiency than wild type and *sae2*Δ cells (Fig 3B), although they did it less efficiently than *mec1*Δ cells, where both Rad53 and Chk1 signaling were abrogated [41]. Strikingly, the lack of Chk1 did not suppress the hypersensitivity to DNA damaging agents of *sae2*Δ cells (Fig 3C), although it overrides the checkpoint-mediated cell cycle arrest.

To rule out the possibility that *CHK1* deletion failed to restore DNA damage resistance in *sae2*Δ cells because it impairs DSB resection, we used JKM139 derivative strains to monitor directly generation of ssDNA at the DSB ends in the absence of Chk1. As ssDNA is resistant to cleavage by restriction enzymes, we followed loss of SspI restriction sites as a measure of resection by Southern blot analysis under alkaline conditions, using a single-stranded probe that anneals to the 3’ end at one side of the break. Consistent with previous indications that Chk1 is not involved in DNA-end resection [38], *chk1*Δ single mutant cells resected the DSB with wild type kinetics (Fig 3D). Furthermore, *CHK1* deletion did not exacerbate the resection defect of *sae2*Δ cells (Fig 3E). Altogether, these data indicate that the prolonged checkpoint-mediated cell cycle arrest of *sae2*Δ cells is not responsible for their hypersensitivity to DNA damaging agents.

### The Rad53-H88Y and Tel1-N2021D variants restore resection and SSA in *sae2*Δ cells

**Fig 4:**
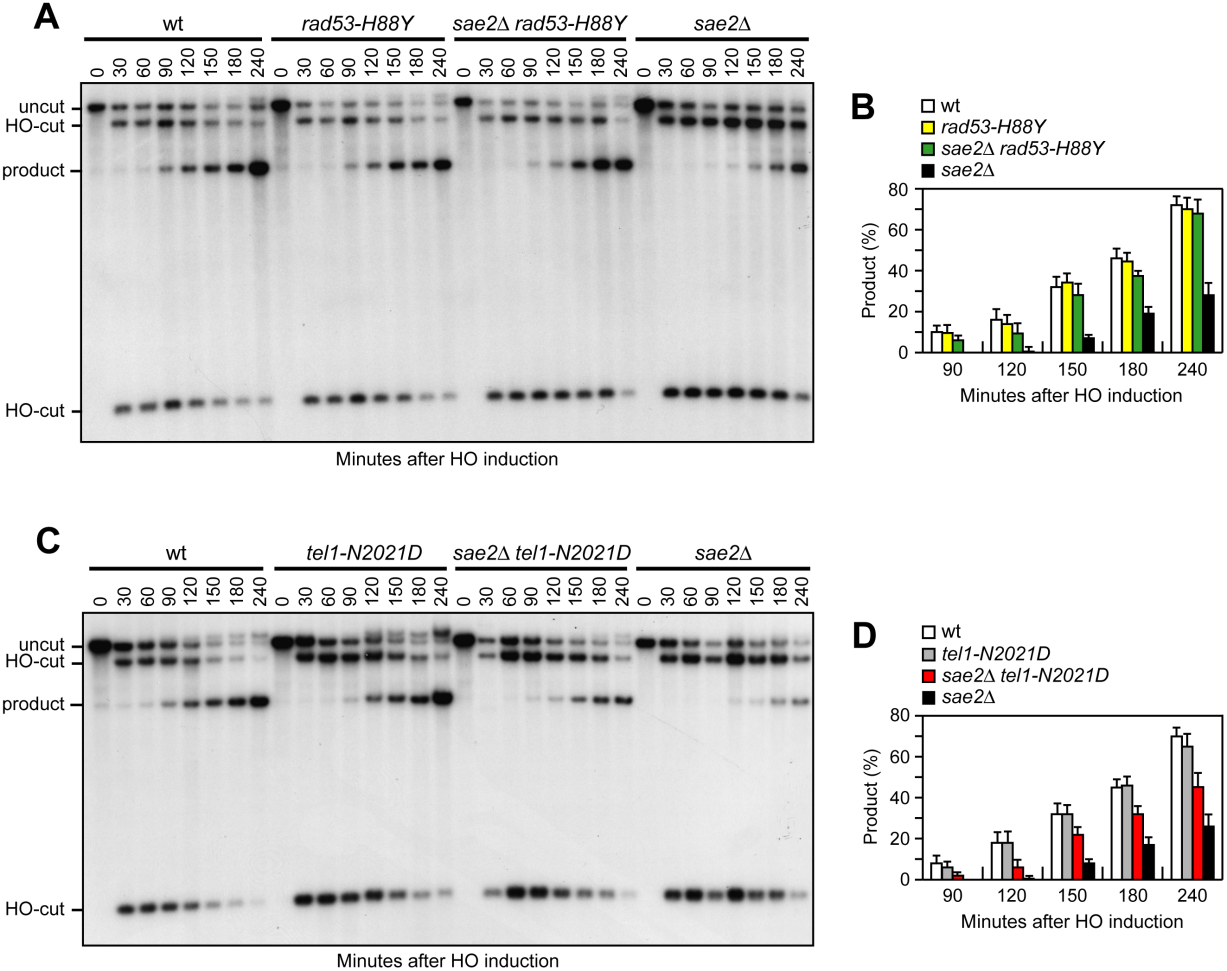
Rad53-H88Y and Tel1-N2021D suppress the SSA defect of *sae2*Δ cells. (A) DSB repair by SSA. YEPR exponentially growing cell cultures of YMV45 derivative strains, carrying the HO-cut site flanked by homologous *leu2* sequences that are 4.6 kb apart, were transferred to YEPRG at time zero. HO-induced DSB formation results in generation of 12 kb and 2.5 kb DNA fragments (HO-cut) that can be detected by Southern blot analysis with a *LEU2* probe of KpnI-digested genomic DNA. DSB repair by SSA generates an 8 kb fragment (product). (B) Densitometric analysis of the product band signals. The experiment as in (A) was independently repeated three times and the mean values are represented with error bars denoting s.d. (n = 3). (C) DSB repair by SSA was analyzed as in (A). (D) Densitometric analysis of the product band signals. The experiment as in (C) was independently repeated three times and the mean values are represented with error bars denoting s.d. (n = 3).

As the checkpoint-mediated cell cycle arrest was not responsible for the DNA damage hypersensitivity of *sae2*Δ cells, we asked whether Rad53-H88Y and/or Tel1-N2021D suppressed the *sae2*Δ resection defect. We first measured the efficiency of single-strand annealing (SSA), a mechanism that repairs a DSB flanked by direct DNA repeats when sufficient resection exposes the complementary DNA sequences, which can then anneal to each other [3]. The *rad53-H88Y* and *tel1-N2021D* alleles were introduced in the YMV45 strain, which carries two tandem *leu2* gene repeats located 4.6 kb apart on chromosome III, with a HO recognition site adjacent to one of the repeats [42]. This strain also harbors a *GAL-HO* construct for galactose-inducible *HO* expression. Both Rad53-H88Y and Tel1-N2021D bypass Sae2 function in SSA-mediated DSB repair. In fact, accumulation of the SSA repair product after HO induction occurred more efficiently in both *sae2*Δ *rad53-H88Y* (Fig 4A and 4B) and *sae2*Δ *tel1-N2021D* (Fig 4C and 4D) than in *sae2*Δ cells, where it was delayed compared to wild type.

**Fig 5:**
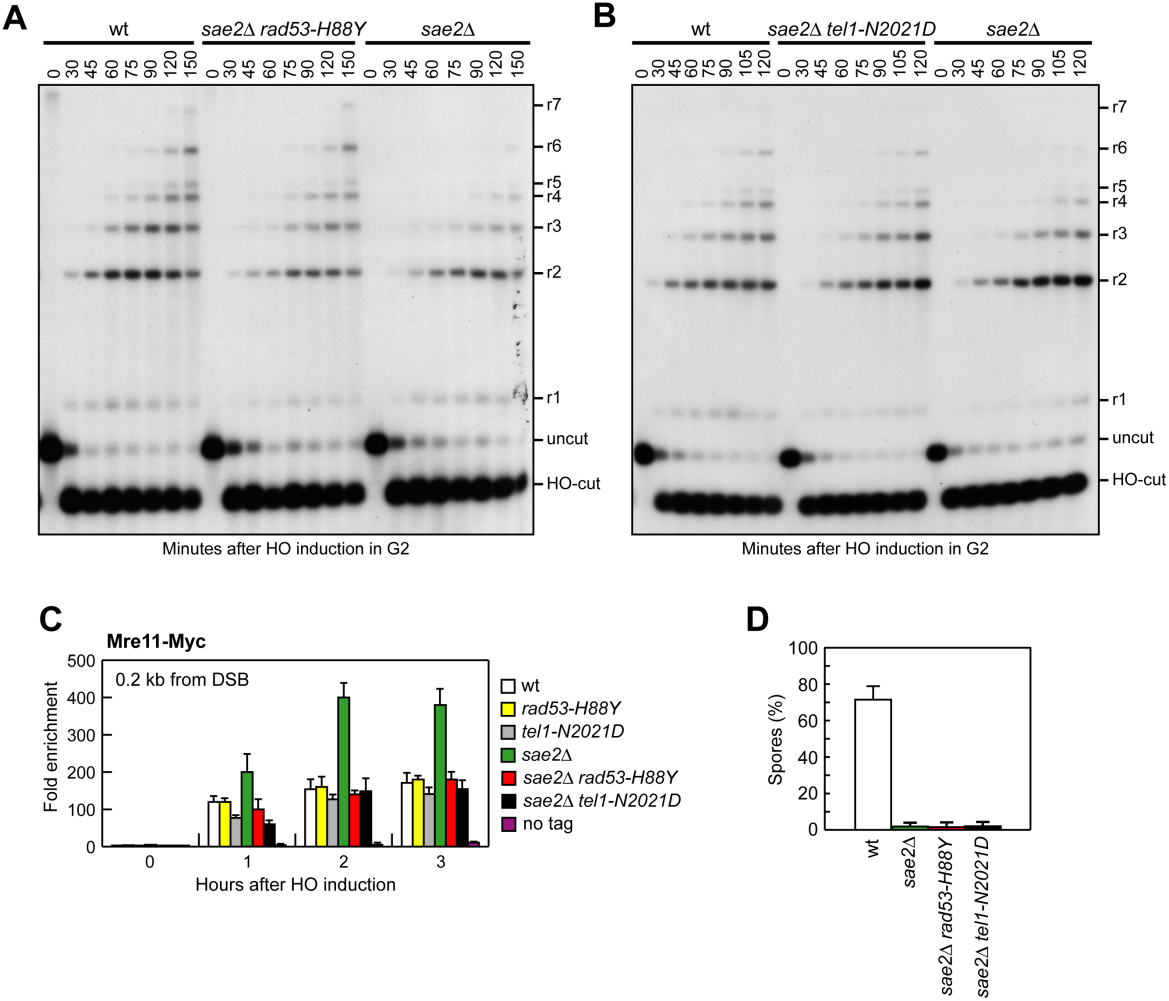
Rad53-H88Y and Tel1-N2021D suppress the resection defect of *sae2*Δ cells. (A, B) DSB resection. YEPR exponentially growing cultures of JKM139 derivative strains were arrested in G2 with nocodazole and transferred to YEPRG in the presence of nocodazole at time zero. Detection of ssDNA was carried out as described in Fig 3D. 5’-3’ resection produces SspI fragments indicated as r1 to r7. (C) Exponentially growing YEPR cell cultures of JKM139 derivative strains were transferred to YEPRG. Relative fold enrichment of Mre11-Myc at 0.2 kb from the HO cleavage site was evaluated after ChIP with anti-Myc antibodies and qPCR analysis compared to untagged Mre11 (no tag). In all diagrams, the ChIP signals were normalized for each time point to the amount of the corresponding input signal. The mean values are represented with error bars denoting s.d. (n = 3). (D) Sporulation efficiency. Spores after 24h in sporulation medium of diploid cells homozygous for the indicated mutations.

To confirm that Rad53-H88Y and Tel1-N2021D suppress the SSA defect of *sae2*Δ cells by restoring DSB resection, we used JKM139 derivative strains to monitor directly generation of ssDNA at the DSB ends. Indeed, *sae2*Δ *rad53-H88Y* (Fig 5A) and *sae2*Δ *tel1-N2021D* (Fig 5B) cells resected the HO-induced DSB more efficiently than *sae2*Δ cells, indicating that both Rad53-H88Y and Tel1-N2021D suppress the resection defect of *sae2*Δ cells.

The DSB resection defect of *sae2*Δ cells is thought to be responsible for the increased persistence of MRX at the DSB [43]. Because Rad53-H88Y and Tel1-N2021D restore DSB resection in *sae2*Δ cells, we expected that the same variants also reduce the amount of MRX bound at the DSB. The amount of Mre11 bound at the HO-induced DSB end turned out to be lower in both *sae2*Δ *rad53-H88Y* and *sae2*Δ *tel1-N2021D* than in *sae2*Δ cells (Fig 5C). Therefore, the Rad53-H88Y and Tel1-N2021D variants restore DSB resection in *sae2*Δ cells and reduce MRX association/persistence at the DSB.

Consistent with the finding that Rad53-H88Y and Tel1-N2021D do not fully restore CPT resistance in *sae2*Δ cells (Fig 1A), and therefore do not bypass completely all Sae2 functions, the *rad53-H88Y* and *tel1-N2021D* mutations were unable to suppress the sporulation defects of *sae2*Δ/*sae2*Δ diploid cells (Fig 5D), suggesting that they cannot bypass the requirement for Sae2/MRX endonucleolytic cleavage to remove Spo11 from meiotic DSBs.

### Suppression of the DNA damage hypersensitivity of *sae2*Δ cells by Rad53-H88Y and Tel1-N2021D variants requires Sgs1-Dna2

**Fig 6:**
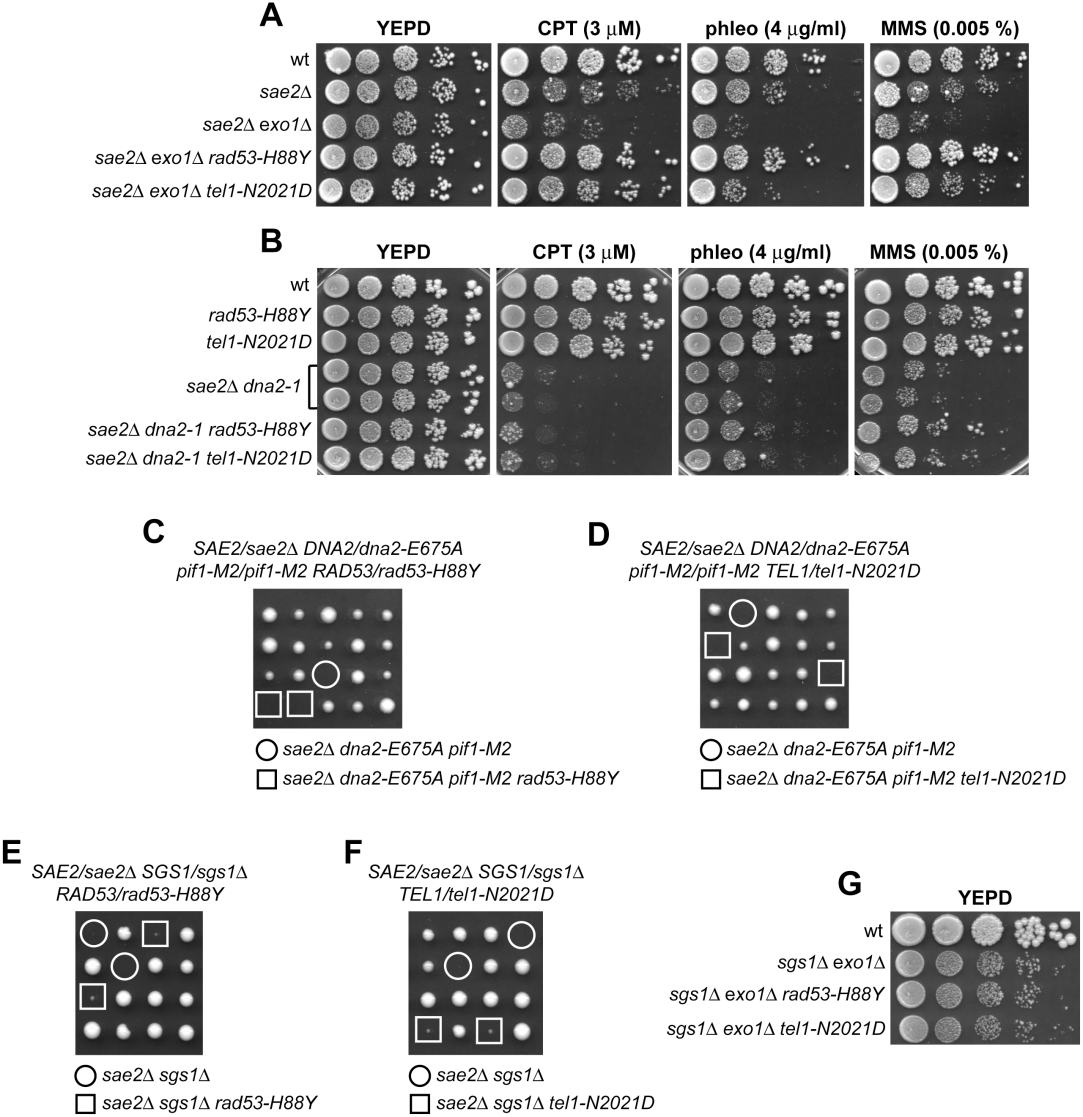
The Rad53-H88Y and Tel1-N2021D bypass of Sae2 function is Sgs1-Dna2-dependent. (A, B) Exponentially growing cells were serially diluted (1:10) and each dilution was spotted out onto YEPD plates with or without CPT, phleomycin or MMS. (C-F) Meiotic tetrads were dissected on YEPD plates that were incubated at 25°C, followed by spore genotyping. (G) Exponentially growing cells were serially diluted (1:10) and each dilution was spotted out onto YEPD plates.

The MRX complex not only provides the nuclease activity for initiation of DSB resection, but also allows extensive resection by promoting the binding at the DSB ends of the resection proteins Exo1 and Sgs1-Dna2 [6,7,10]. Suppression of the DNA damage hypersensitivity of *sae2*Δ cells by Rad53-H88Y and Tel1-N2021D requires the physical presence of the MRX complex but not its nuclease activity (Fig 1C and 1D). As the loading of Exo1, Sgs1-Dna2 at DSBs depends on the MRX complex independently of its nuclease activity [10], we asked whether the investigated suppression events require Exo1, Sgs1 and/or Dna2. This question was particularly interesting, as Rad53 was shown to inhibit resection at uncapped telomeres through phosphorylation and inhibition of Exo1 [38,44]. As shown in Fig 6A, *sae2*Δ suppression by Rad53-H88Y and Tel1-N2021D was Exo1-independent. In fact, although the lack of Exo1 exacerbated the sensitivity to DNA damaging agents of *sae2*Δ cells, both *sae2*Δ *exo1*Δ *rad53-H88Y* and *sae2*Δ *exo1*Δ *tel1-N2021D* triple mutants were more resistant to genotoxic agents than *sae2*Δ *exo1*Δ double mutant cells (Fig 6A).

By contrast, neither Rad53-H88Y nor Tel1-N2021D were able to suppress the sensitivity to DNA damaging agents of *sae2*Δ cells carrying the temperature sensitive *dna2-1* allele (Fig 6B), suggesting that Dna2 activity is required for their suppressor effect. Dna2, in concert with the helicase Sgs1, functions as a nuclease in DSB resection [7]. The *dna2-E675A* allele abolishes Dna2 nuclease activity, which is essential for cell viability and whose requirement is bypassed by the *pif1-M2* mutation that impairs the nuclear activity of the Pif1 helicase [45]. The lack of Sgs1 or expression of the Dna2-E675A variant in the presence of the *pif1-M2* allele impaired viability of *sae2*Δ cells even in the absence of genotoxic agents. The synthetic lethality of *sae2*Δ *sgs1*Δ cells, and possibly of *sae2*Δ *dna2-E675A pif1-M2*, is likely due to defects in DSB resection, as it is known to be suppressed by either *EXO1* overexpression or *KU* deletion [11]. Thus, we asked whether Rad53-H88Y and/or Tel1-N2021D could restore viability of *sae2*Δ *sgs1*Δ and/or *sae2*Δ *dna2-E675A pif1-M2* cells. Tetrad dissection of diploid cells did not allow to find viable spores with the *sae2*Δ *dna2-E675A pif1-M2 rad53-H88Y* (Fig 6C) or *sae2*Δ *dna2-E675A pif1-M2 tel1-N2021D* genotypes (Fig 6D), indicating that neither Rad53-H88Y nor Tel1-N2021D can restore the viability of *sae2*Δ *dna2-E675A pif1-M2* cells. Similarly, no viable *sae2*Δ *sgs1*Δ spores could be recovered, while *sae2*Δ *sgs1*Δ *rad53-H88Y* and *sae2*Δ *sgs1*Δ *tel1-N2021D* triple mutant spores formed very small colonies that could not be further propagated (Fig 6E and 6F). Finally, neither Rad53-H88Y nor Tel1-N2021D, which allowed DNA damage resistance in *sae2*Δ *exo1*Δ cells (Fig 6A), were able to suppress the growth defect of *sgs1*Δ *exo1*Δ double mutant cells even in the absence of genotoxic agents (Fig 6G). Altogether, these findings indicate that suppression by Rad53-H88Y and Tel1-N2021D of the DNA damage hypersensitivity caused by the absence of Sae2 is dependent on Sgs1-Dna2.

### The lack of Rad53 kinase activity suppresses the DNA damage hypersensitivity and the resection defect of *sae2*Δ cells

**Fig 7:**
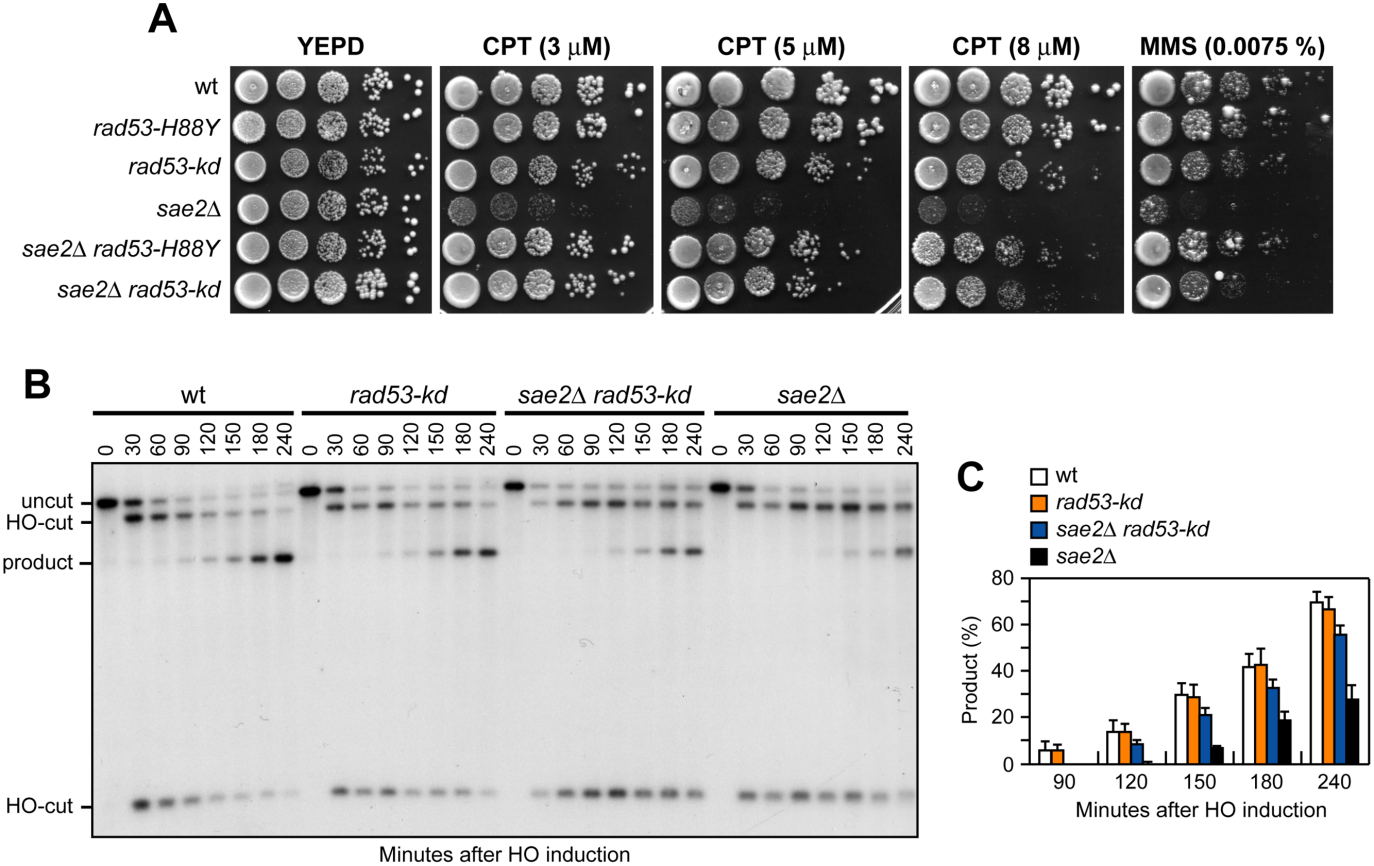
The Rad53-kd variant restores DNA damage resistance and SSA in *sae2*Δ cells. (A) Exponentially growing cells were serially diluted (1:10) and each dilution was spotted out onto YEPD plates with or without CPT or MMS. (B) DSB repair by SSA. The analysis was performed as described in Fig 4A. (C) Densitometric analysis of the product band signals. The experiment as in (B) was independently repeated three times and the mean values are represented with error bars denoting s.d. (n = 3).

The Rad53-H88Y protein is defective in interaction with Rad9 (Fig 2C) and therefore fails to undergo autophosphorylation and activation, prompting us to test whether other mutations affecting Rad53 activity can bypass Sae2 functions. To this end, we could not use *rad53*Δ cells because they show growth defects even when the lethal effect of *RAD53* deletion is suppressed by the lack of Sml1 [46]. We then substituted the chromosomal wild type *RAD53* allele with the kinase-defective *rad53-K227A* allele (*rad53-kd*), which does not impair cell viability in the absence of genotoxic agents but affects checkpoint activation [47]. The *rad53-kd* allele rescued the sensitivity of *sae2*Δ cells to CPT and MMS to an extent similar to Rad53-H88Y (Fig 7A). Furthermore, accumulation of the SSA repair products occurred more efficiently in *sae2*Δ *rad53-kd* cells than in *sae2*Δ (Fig 7B and 7C), indicating that the lack of Rad53 kinase activity bypasses Sae2 function in SSA-mediated DSB repair.

### The lack of Tel1 kinase activity bypasses Sae2 function at DSBs, whereas Tel1 hyperactivation increases Sae2 requirement

**Fig 8:**
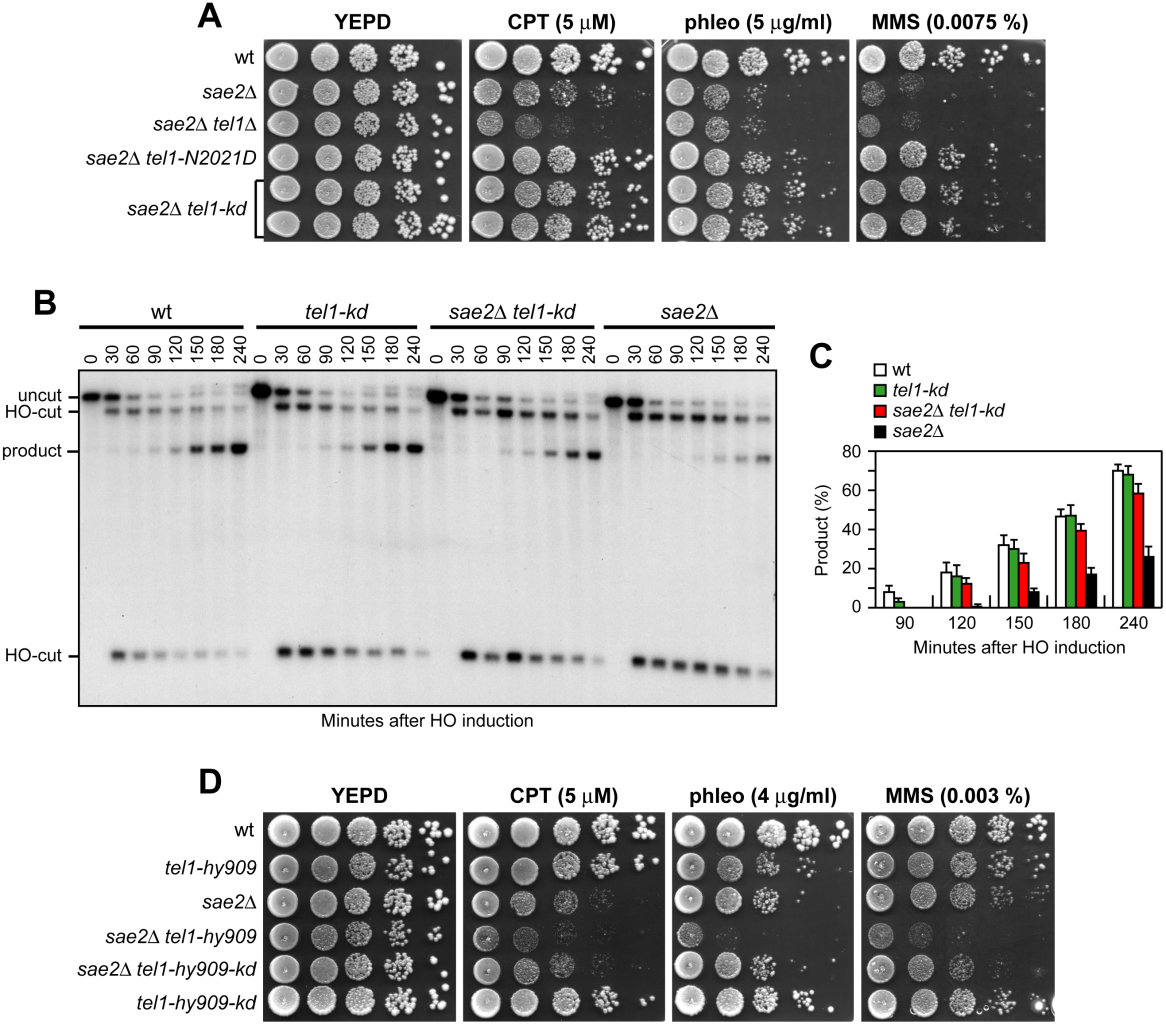
The Tel1-kd variant restores DNA damage resistance and SSA in *sae2*Δ cells. (A) Exponentially growing cells were serially diluted (1:10) and each dilution was spotted out onto YEPD plates with or without CPT, phleomycin or MMS. (B) DSB repair by SSA. The analysis was performed as described in Fig 4A. (C) Densitometric analysis of the product band signals. The experiment as in (B) was independently repeated three times and the mean values are represented with error bars denoting s.d. (n = 3). (D) Exponentially growing cells were serially diluted (1:10) and each dilution was spotted out onto YEPD plates with or without CPT, phleomycin or MMS.

Suppression of *sae2*Δ may be peculiar to Tel1-N2021D, which is poorly recruited to DSBs (Fig 2F), or it might be performed also by *TEL1* deletion (*tel1*Δ) or by expression of a Tel1 kinase defective variant (Tel1-kd). Indeed, the Tel1-kd variant, carrying the G2611D, D2612A, N2616K, and D2631E amino acid substitutions that abolish Tel1 kinase activity in vitro (Fig 2E) [35], rescued the hypersensitivity of *sae2*Δ cells to genotoxic agents to an extent similar to Tel1-N2021D (Fig 8A). The lack of Tel1 kinase activity bypassed also Sae2 function in DSB resection, because *sae2*Δ *tel1-kd* cells repaired a DSB by SSA more efficiently than *sae2*Δ cells (Fig 8B and 8C). By contrast, and consistent with previous studies [23,24], *TEL1* deletion was not capable to suppress the hypersensitivity to DNA damaging agents of *sae2*Δ cells (Fig 8A). Rather, *tel1*Δ *sae2*Δ double mutant cells displayed higher sensitivity to CPT than *sae2*Δ cells (Fig 8A). Altogether, these data indicate that the lack of Tel1 kinase activity can bypass Sae2 function both in DNA damage resistance and DSB resection, but these suppression events require the physical presence of the Tel1 protein.

As impairment of Tel1 function rescued the *sae2*Δ defects, we asked whether Tel1 hyperactivation exacerbates the DNA damage hypersensitivity of *sae2*Δ cells. We previously isolated the *TEL1-hy909* allele, which encodes a Tel1 mutant variant with enhanced kinase activity that causes an impressive telomere overelongation [48]. As shown in Fig 8D, *sae2*Δ *TEL1-hy909* double mutant cells were more sensitive to DNA damaging agents than *sae2*Δ single mutant cells. This enhanced DNA damage sensitivity was likely due to Tel1 kinase activity, as *sae2*Δ cells expressing a kinase defective Tel1-hy909-kd variant were as sensitive to DNA damaging agents as *sae2*Δ cells (Fig 8D). Thus, impairment of Tel1 activity bypasses Sae2 function at DSBs, whereas Tel1 hyperactivation increases the requirement for Sae2 in survival to genotoxic stress.

**Fig 9:**
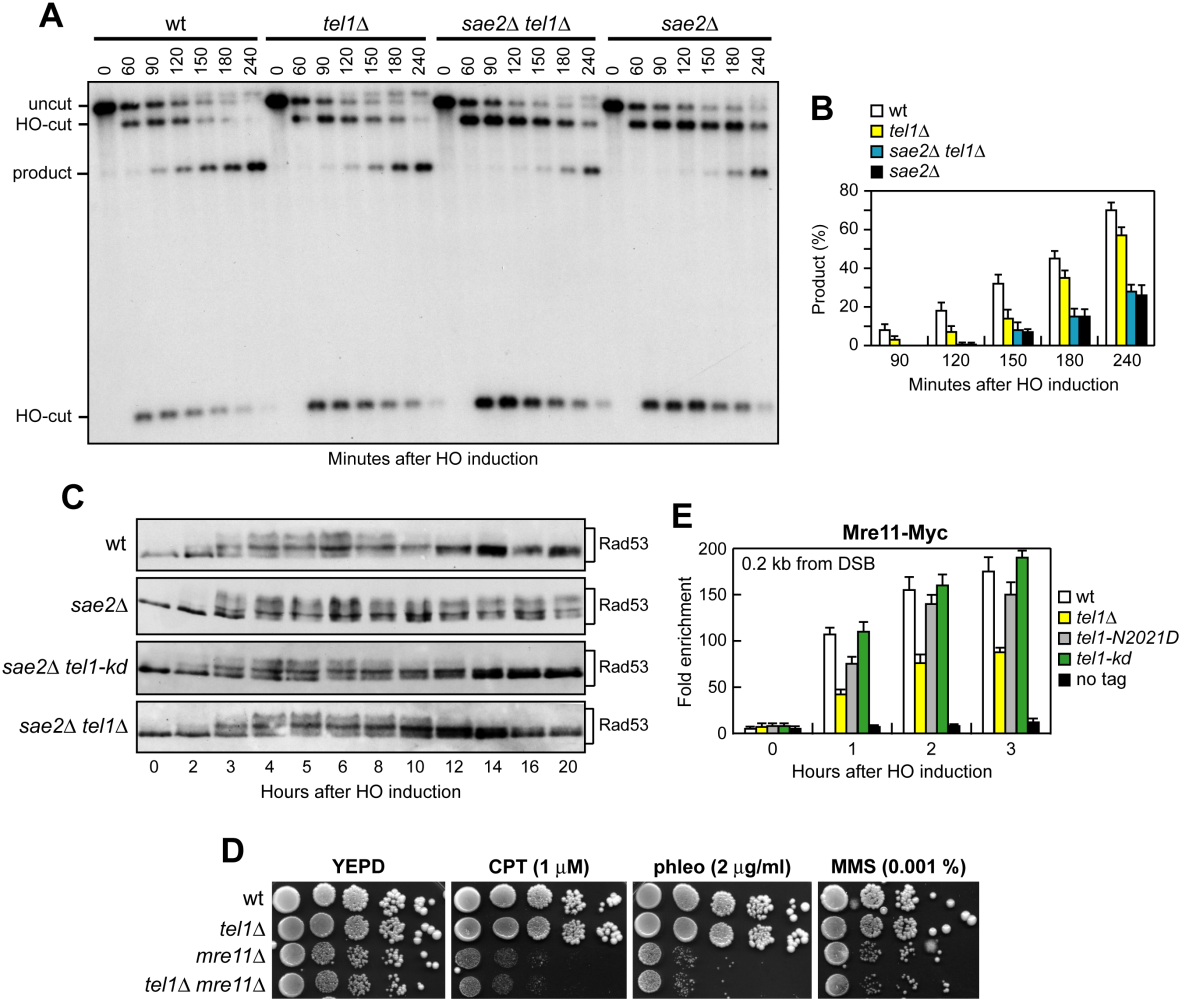
The lack of Tel1 does not restore DNA damage resistance and SSA in *sae2*Δ cells. (A) DSB repair by SSA. The analysis was performed as described in Fig 4A. (B) Densitometric analysis of the product band signals. The experiment as in (A) was independently repeated three times and the mean values are represented with error bars denoting s.d. (n = 3). (C) Exponentially growing YEPR cell cultures of JKM139 derivative strains were transferred to YEPRG (time zero), followed by western blot analysis with anti-Rad53 antibodies of protein extracts prepared at the indicated time points. (D) Exponentially growing cells were serially diluted (1:10) and each dilution was spotted out onto YEPD plates with or without CPT, phleomycin or MMS. (E) ChIP analysis. Exponentially growing YEPR cell cultures of JKM139 derivative strains were transferred to YEPRG. Recruitment of Mre11-Myc compared to untagged Mre11 (no tag) at 0.2 kb from the HO-cut was determined by ChIP analysis and qPCR. In all diagrams, the ChIP signals were normalized for each time point to the amount of the corresponding input signal. The mean values are represented with error bars denoting s.d. (n = 3).

The absence of Tel1 failed not only to restore DNA damage resistance in *sae2*Δ cells (Fig 8A), but also to suppress their SSA defect (Fig 9A and 9B). The difference in the effects of *tel1*Δ and *tel1-kd* was not due to checkpoint signaling, as Rad53 phosphorylation decreased with similar kinetics in both *sae2*Δ *tel1-kd* and *sae2*Δ *tel1*Δ double mutant cells 10–12 hours after HO induction (Fig 9C). Interestingly, SSA-mediated DSB repair occurred with wild type kinetics in *tel1-kd* mutant cells (Fig 8B and 8C), while *tel1*Δ cells repaired a DSB by SSA less efficiently than wild type cells (Fig 9A and 9B), suggesting that Tel1 might have a function at DSBs that does not require its kinase activity. Indeed, *TEL1* deletion was shown to slight impair DSB resection [19]. Furthermore, it did not exacerbate the resection defect [19] and the hypersensitivity to DNA damaging agents of *mre11*Δ cells (Fig 9D), suggesting that the absence of Tel1 can impair MRX function. Tel1 was also shown to promote MRX association at DNA ends flanked by telomeric DNA repeats independently of its kinase activity [49], and we are showing that suppression of *sae2*Δ by Tel1-N2021D requires the physical presence of the MRX complex (Fig 1D). Thus, it is possible that the lack of Tel1 fails to bypass Sae2 function at DSBs because it reduces MRX association at DSBs to a level that is not sufficient to restore DNA damage resistance and DSB resection in *sae2*Δ cells. Indeed, the amount of Mre11 bound at the HO-induced DSB was decreased in *tel1*Δ, but not in *tel1-kd* cells, compared to wild type (Fig 9E). In agreement with a partial loss of Tel1 function, the Tel1-N2021D variant, whose association to DSBs is diminished compared to wild type Tel1 but not abolished (Fig 2F), only slightly decreased Mre11 association to the DSB (Fig 9E). As the rescue of *sae2*Δ by Tel1-N2021D requires the physical presence of the MRX complex, this Tel1 function in promoting MRX association to DSBs can explain the inability of *tel1*Δ to bypass Sae2 function in DNA damage resistance and resection.

### Tel1 and Rad53 kinase activities promote Rad9 binding to the DSB ends

**Fig 10:**
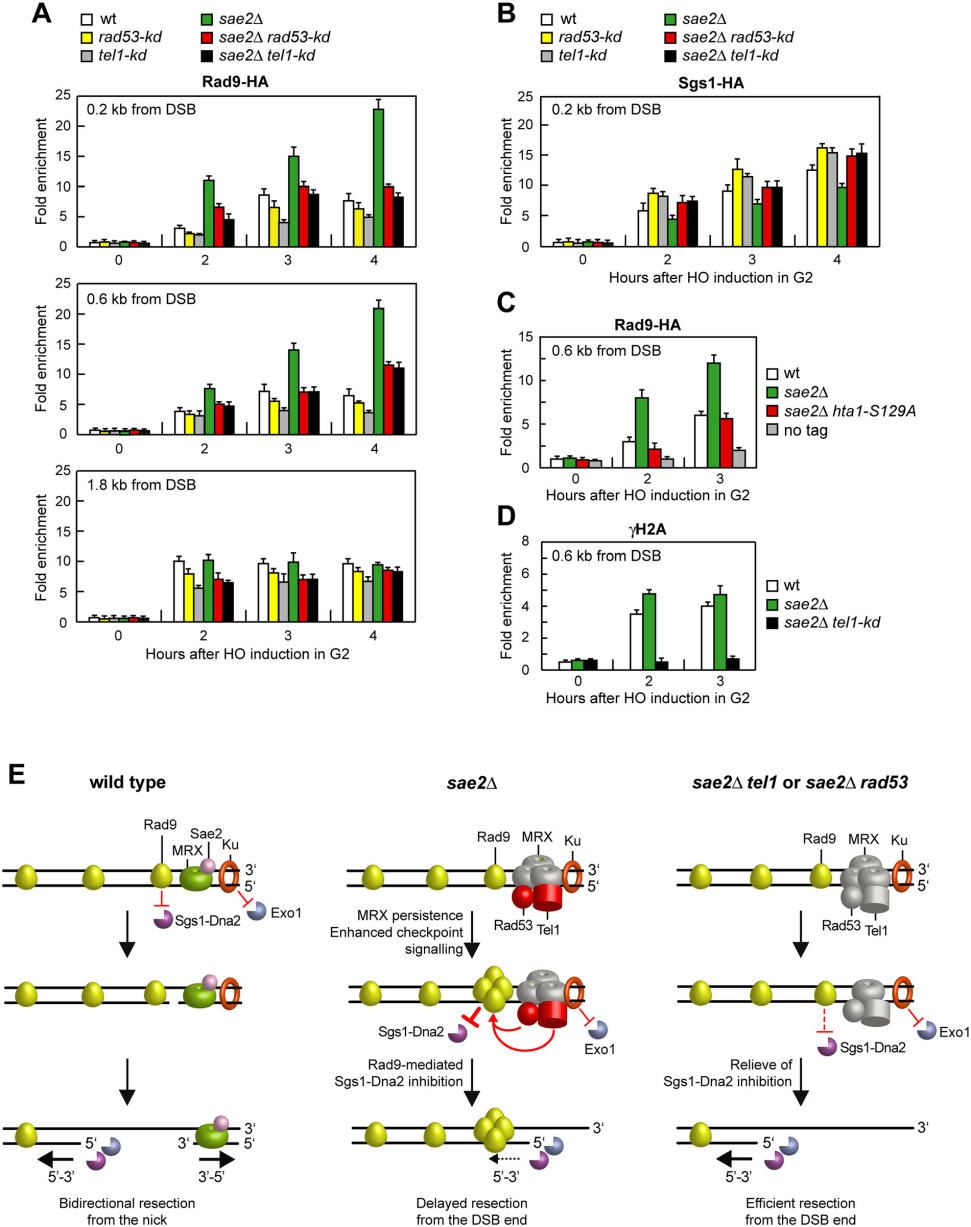
Rad53-kd and Tel1-kd prevent Rad9 association at DSBs. (A) Exponentially growing YEPR cell cultures of JKM139 derivative strains were arrested in G2 with nocodazole and transferred to YEPRG in the presence of nocodazole. Recruitment of Rad9-HA at the indicated distance from the HO-cut was determined by ChIP and qPCR. In all diagrams, the ChIP signals were normalized for each time point to the amount of the corresponding input signal. The mean values are represented with error bars denoting s.d. (n = 3). (B) As in (A), but showing Sgs1-HA binding. (C) As in (A). All strains carried also the deletion of *HTA2* gene. (D) As in (A), but showing γH2A binding. (E) Model for the role of Sae2 at DSBs. (Left) Sae2 activates the Mre11 endonuclease activity to incise the 5’ strand. Generation of the nick allows bidirectional processing by Exo1/Sgs1-Dna2 in the 5’-3’ direction from the nick and MRX in the 3’ to 5’ direction toward the DSB ends. Ku and Rad9 inhibit DSB resection by limiting Exo1 and Sgs1-Dna2, respectively. (Middle) The absence of Sae2 impairs the MRX nuclease activity (non functional MRX nuclease is in grey). As a consequence, the endonucleolytic cleavage of the 5’ strand does not occur and resection is carried out by Exo1 and Dna2-Sgs1 that degrade the 5’ strands from the DSB ends. Impairment of Mre11 nuclease activity also causes increased MRX association at the DSB, which leads to enhanced Tel1-dependent Rad53 activation. Tel1 and Rad53 activities limit DSB resection from the DSB end (dashed arrow) by increasing the amount of DSB-bound Rad9, which inhibits Sgs1-Dna2 recruitment at DSBs. (Right) Impairments of Tel1 or Rad53 activity (non functional Tel1 and Rad53 are in grey) restore efficient resection in *sae2*Δ cells by relieving Rad9-mediated inhibition of Sgs1-Dna2. Restored DSB resection by Sgs1-Dna2 also reduces MRX persistence at the DSB.

The suppression of the DNA damage hypersensitivity of *sae2*Δ cells by Rad53-H88Y and Tel1-N2021D requires Dna2-Sgs1 (Fig 6B–6G). Because Sgs1-Dna2 activity is counteracted by Rad9, whose lack restores DSB resection in *sae2*Δ cells [13,14], we asked whether suppression of the DSB resection defect of *sae2*Δ cells by Rad53 or Tel1 dysfunction might be due to decreased Rad9 association to the DSB ends. We have previously shown that wild type and *sae2*Δ cells have similar amounts of Rad9 bound at 1.8 kb from the DSB (Fig 10A) [43]. However, a robust increase in the amount of Rad9 bound at 0.2 kb and 0.6 kb from the DSB was detected in *sae2*Δ cells compared to wild type (Fig 10A) [14]. Strikingly, this enhanced Rad9 accumulation in *sae2*Δ cells was reduced in the presence of the Rad53-kd or Tel1-kd variant, which both decreased the amount of Rad9 bound at the DSB also in otherwise wild type cells (Fig 10A). Thus, Rad9 association close to the DSB depends on Rad53 and Tel1 kinase activity.

Rad9 inhibits DSB resection by counteracting Sgs1 recruitment to DSBs [13] and, as expected, Sgs1 binding to DSBs was lower in *sae2*Δ cells than in wild type (Fig 10B). By contrast, the presence of Rad53-kd or Tel1-kd variants increased the amount of Sgs1 at the DSB in both wild type and *sae2*Δ cells (Fig 10B). Together with the observation that the suppression of *sae2*Δ hypersensitivity to genotoxic agents by Rad53 and Tel1 dysfunctions requires Sgs1-Dna2, these findings indicate that the lack of Rad53 or Tel1 kinase activity restores DSB resection in *sae2*Δ cells by decreasing Rad9 association close to the DSB and therefore by relieving Sgs1-Dna2 inhibition. Although both *rad53-kd* and *tel1-kd* cells showed some lowering of Rad9 binding at DSBs compared to wild type cells (Fig 10A), they did not appear to accelerate SSA, suggesting that this extent of Rad9 binding is anyhow sufficient to limit resection in a wild type context.

Rad9 is known to be enriched at the sites of damage by interaction with histone H2A that has been phosphorylated on serine 129 (γH2A) by Mec1 and Tel1 [50–53]. As the lack of γH2A suppresses the SSA defect of *sae2*Δ cells [14], Tel1 activity might increase the amount of Rad9 bound at the DSB in *sae2*Δ cells by promoting generation of γH2A. Indeed, the *hta1-S129A* allele, which encodes a H2A variant where Ser129 is replaced by a non-phosphorylatable alanine residue, thus causing the lack of γH2A, suppressed the resection defect of *sae2*Δ cells (S3 Fig). Furthermore, γH2A formation turned out to be responsible for the enhanced Rad9 binding close to the break site, as *sae2*Δ *hta1-S129A* cells showed wild type levels of Rad9 bound at the DSB (Fig 10C). Finally, γH2A formation close to the DSB depends on Tel1 kinase activity, as γH2A at the DSB was not detectable in *sae2*Δ *tel1-kd* cells (Fig 10D). Altogether, these data indicate that Tel1 promotes Rad9 association to DSB in *sae2*Δ cells through γH2A generation.

## Discussion

Cells lacking Sae2 not only are defective in DSB resection, but also show persistent DSB-induced checkpoint activation that causes a prolonged cell cycle arrest. This enhanced checkpoint signaling is due to persistent MRX binding at the DSBs, which activates a Tel1-dependent checkpoint that is accompanied by Rad53 phosphorylation [20,22]. While failure to remove MRX from the DSBs has been shown to sensitize *sae2*Δ cells to genotoxic agents [23,24], the possible contribution of the DNA damage checkpoint in determining the DNA damage hypersensitivity and the resection defect of *sae2*Δ cells has never been studied in detail.

We show that impairment of Rad53 activity either by affecting its interaction with Rad9 (Rad53-H88Y) or by abolishing its kinase activity (Rad53-kd) suppresses the sensitivity to DNA damaging agents of *sae2*Δ cells. A similar effect can be detected also when Tel1 function is compromised either by reducing its recruitment to DSBs (Tel1-N2021D) or by abrogating its kinase activity (Tel1-kd). These suppression effects are not due to the escape of the checkpoint-mediated cell cycle arrest, as *CHK1* deletion, which overrides the persistent cell cycle arrest of *sae2*Δ cells, does not suppress the hypersensitivity of the same cells to DNA damaging agents. Rather, we found that impairment of Rad53 or Tel1 signaling suppresses the resection defect of *sae2*Δ by decreasing the amount of Rad9 bound very close to the break site. As it is known that Rad9 inhibits Sgs1-Dna2 [13,14], this reduced Rad9 association at DSBs relieves inhibition of Sgs1-Dna2 activity that can then compensate for the lack of Sae2 function in DSB resection. In this view, active Rad53 and Tel1 increase the requirement for Sae2 in DSB resection by promoting Rad9 binding to DSBs and therefore by inhibiting Sgs1-Dna2. Consistent with a role of Sgs1 in removing MRX from the DSBs [54], the relieve of Sgs1-Dna2 inhibition by Rad53 or Tel1 dysfunction leads to a reduction of MRX association to DSBs in *sae2*Δ cells.

Our finding that Tel1 or Rad53 inactivation can restore both DNA damage resistance and DSB resection in *sae2*Δ cells is apparently at odds with previous findings that attenuation of the Rad53-dependent checkpoint signaling by decreasing MRX association to DSBs suppresses the DNA damage hypersensitivity of *sae2*Δ cells but not their resection defect [23,24]. Noteworthy, the bypass of Sae2 function by Rad53 or Tel1 dysfunction requires the physical presence of MRX bound at DSBs, which is known to promote stable association of Exo1, Sgs1 and Dna2 to DSBs [10]. Thus, we speculate that a reduced MRX association at DSBs allows *sae2*Δ cells to initiate DSB resection by relieving Rad9-mediated inhibition of Sgs1-Dna2 activity. As DSB repair by HR has been shown to require limited amount of ssDNA at DSB ends [55,56], the ssDNA generated by this initial DSB processing might be sufficient to restore DNA damage resistance in *sae2*Δ cells even when wild type levels of resection are not restored because DSB-bound MRX is not enough to ensure stable Sgs1 and Dna2 association.

Surprisingly, *TEL1* deletion, which relieves the persistent Tel1-dependent checkpoint activation caused by the lack of Sae2, did not restore DNA damage resistance and DSB resection in *sae2*Δ cells. We found that the lack of Tel1 protein affects the association of MRX to the DSB ends independently of its kinase activity. As the rescue of *sae2*Δ by Tel1-N2021D requires the physical presence of the MRX complex, this reduced MRX-DNA association can explain the inability of *TEL1* deletion to restore DNA damage resistance and resection in *sae2*Δ cells. Therefore, while an enhanced Tel1 signaling activity in the absence of Sae2 leads to DNA damage hypersensitivity and resection defects, a sufficient amount of Tel1 needs to be present at DSBs to support MRX function at DSBs.

How do Rad53 and Tel1 control Rad9 association to DSB? Rad53-mediated phosphorylation of Rad9 does not appear to promote Rad9 binding to the DSB [57,58]. Because Rad53 and RPA compete for binding to Sgs1 [59], it is tempting to propose that impaired Rad53 signaling activity might shift Sgs1 binding preference from Rad53 to RPA, leading to increased Sgs1 association to RPA-coated DNA that can counteract Rad9 binding and inhibition of resection. In turn, Tel1 and Mec1 can phosphorylate Rad9 [60,61], and abrogation of these phosphorylation events rescues the sensitivity to DNA damaging agents of *sae2*Δ cells [14], suggesting that Tel1 might control Rad9 association to DSBs directly through phosphorylation. On the other hand, Tel1 promotes generation of γH2A [50–53], which counteracts DSB resection by favoring Rad9 association at the DSB [43]. We show that expression of a non-phosphorylatable H2A variant in *sae2*Δ cells suppresses their resection defect and prevents the accumulation of Rad9 at the DSB. Furthermore, γH2A generation close to the break site depends on Tel1 kinase activity. Thus, although we cannot exclude a direct control of Tel1 on Rad9 association to DNA ends, our findings indicate that Tel1 acts in this process mostly through γH2A generation.

Altogether, our results support a model whereby Tel1 and Rad53, once activated, limit DSB resection by promoting Rad9 binding to DSBs and therefore by inhibiting Sgs1-Dna2. Sae2 activates Mre11 endonucleolytic activity that clips the 5’-terminated DNA strand, thus generating 5’ and 3’ tailed substrates that can be processed by Exo1/Sgs1-Dna2 and Mre11 activity, respectively (Fig 10E, left). When Sae2 function fails, defective Mre11 nuclease activity causes increased MRX persistence at the DSB that leads to enhanced and prolonged Tel1-dependent Rad53 activation. As a consequence, Tel1- and Rad53-mediated phosphorylation events increase the amount of Rad9 bound at the DSB, which inhibits DSB resection by counteracting Sgs1-Dna2 activity (Fig 10E, middle). Dysfunction of Rad53 or Tel1 reduces Rad9 recruitment at the DSB ends and therefore relieves inhibition of Sgs1-Dna2, which can compensate for the lack of Sae2 in DNA damage resistance and resection (Fig 10E, right). Altogether, these findings indicate that the primary cause of the resection defect of *sae2*Δ cells is an enhanced Rad9 binding to DSBs that is promoted by the persistent MRX-dependent Tel1 and Rad53 signaling activities.

ATM inhibition has been proposed as a strategy for cancer treatment [62]. Therefore, the observation that dampening Tel1/ATM signaling activity restores DNA damage resistance in *sae2*Δ cells might have implications in cancer therapies that use ATM inhibitors for synthetic lethal approaches to threat tumors with deficiencies in the DNA damage response.

## Materials and Methods

### Yeast strains

The yeast strains used in this study are derivatives of W303, JKM139 and YMV45 strains and are listed in S1 Table. Cells were grown in YEP medium (1% yeast extract, 2% peptone) supplemented with 2% glucose (YEPD), 2% raffinose (YEPR) or 2% raffinose and 3% galactose (YEPRG).

### Search for suppressors of *sae2*Δ sensitivity to CPT

To search for suppressor mutations of the CPT-sensitivity of *sae2*Δ mutant, 5x10^6^ *sae2*Δ cells were plated on YEPD in the presence of 30μM CPT. Survivors were crossed to wild type cells to identify by tetrad analysis the suppression events that were due to single-gene mutations. Genomic DNA from two single-gene suppressors was analyzed by next-generation Illumina sequencing (IGA technology services) to identify mutations altering open reading frames within the reference *S*. *cerevisiae* genome. To confirm that *rad53-H88Y* and *tel1-N2021D* mutations were responsible for the suppression, either *URA3* or *HIS3* gene was integrated downstream of the *rad53-H88Y* and *tel1-N2021D* stop codon, respectively, and the resulting strain was crossed to wild type cells to verify by tetrad dissection that the suppression of the *sae2*Δ CPT sensitivity co-segregated with the *URA3* or *HIS3* allele.

### DSB resection and repair by SSA

DSB end resection at the *MAT* locus in JKM139 derivative strains was analyzed on alkaline agarose gels as previously described [63]. DSB formation and repair in YMV45 strain were detected by Southern blot analysis using an *Asp*718-*Sal*I fragment containing part of the *LEU2* gene as a probe as previously described [63]. Quantitative analysis of the repair product was performed by calculating the ratio of band intensities for SSA product with respect to a loading control.

### Other techniques

Protein extracts for western blot analysis were prepared by TCA precipitation. ChIP assays were performed as previously described [64]. Data are expressed as fold enrichment at the HO-induced DSB over that at the non-cleaved *ARO1* locus, after normalization of each ChIP signals to the corresponding amount of immunoprecipitated protein and input for each time point. Fold enrichment was then normalized to the efficiency of DSB induction. The kinase assay and coimmunoprecipitation were performed as previously described [48]. Rad53 was detected by using anti-Rad53 polyclonal antibodies (ab104232) from Abcam. γH2A was immunoprecipitated by using anti-γH2A antibodies (ab15083) from Abcam.

## Supporting Information

S1 Fig *rad53-H88Y* and *tel1-N2021D* suppressor alleles are recessive. Exponentially growing cells were serially diluted (1:10) and each dilution was spotted out onto YEPD plates with or without the indicated genotoxic agents.
(TIF)

S2 Fig The Tel1-N2021D variant does not affect telomere length. Genomic DNA prepared from exponentially growing cells was digested with *Xho*I and hybridized with a poly(GT) telomere-specific probe.
(TIF)

S3 Fig The lack of γH2A suppresses the resection defect of *sae2*Δ cells. DSB resection. YEPR exponentially growing cultures of JKM139 derivative cells with the indicated genotypes were arrested in G2 with nocodazole and transferred to YEPRG in the presence of nocodazole at time zero. All strains carried also the deletion of *HTA2* gene. Gel blots of SspI-digested genomic DNA separated on alkaline agarose gel were hybridized with a single-stranded RNA probe that anneals to the unresected strand on one side of the break. 5’-3’ resection progressively eliminates SspI sites, producing larger SspI fragments (r1 through r7) detected by the probe.
(TIF)

S1 Table List of yeast strains described in this work. (DOC)
