# Supplementary figures and images for: Sae2 Function at DNA Double-Strand Breaks Is Bypassed by Dampening Tel1 or Rad53 Activity
Source: PLoS Genet. 2015 Nov 19;11(11):e1005685. doi: 10.1371/journal.pgen.1005685 (PMC4652893; doi:10.1371/journal.pgen.1005685)

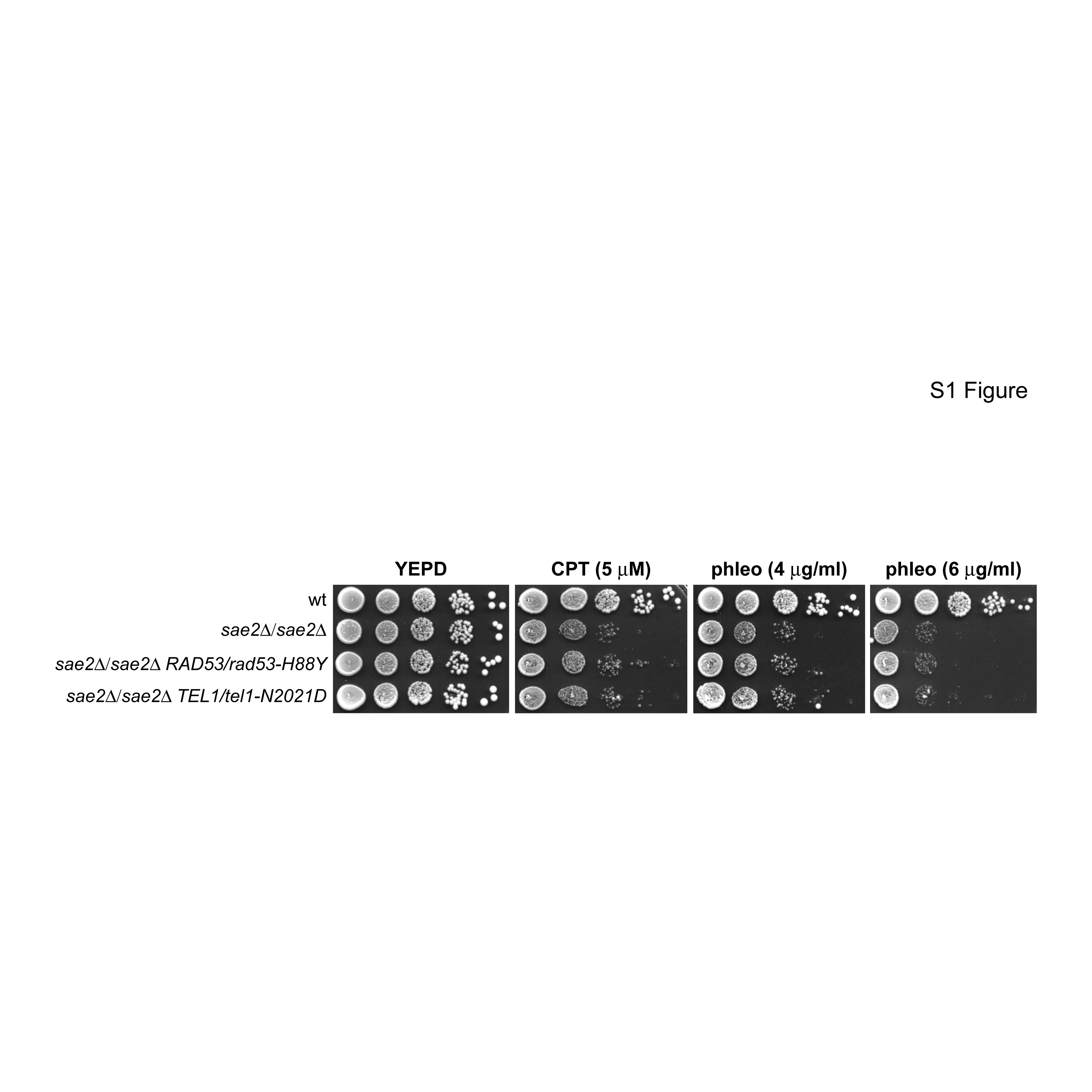

Supplement: S1 Fig — Exponentially growing cells were serially diluted (1:10) and each dilution was spotted out onto YEPD plates with or without the indicated genotoxic agents. (TIF) [file pgen.1005685.s001.tif]

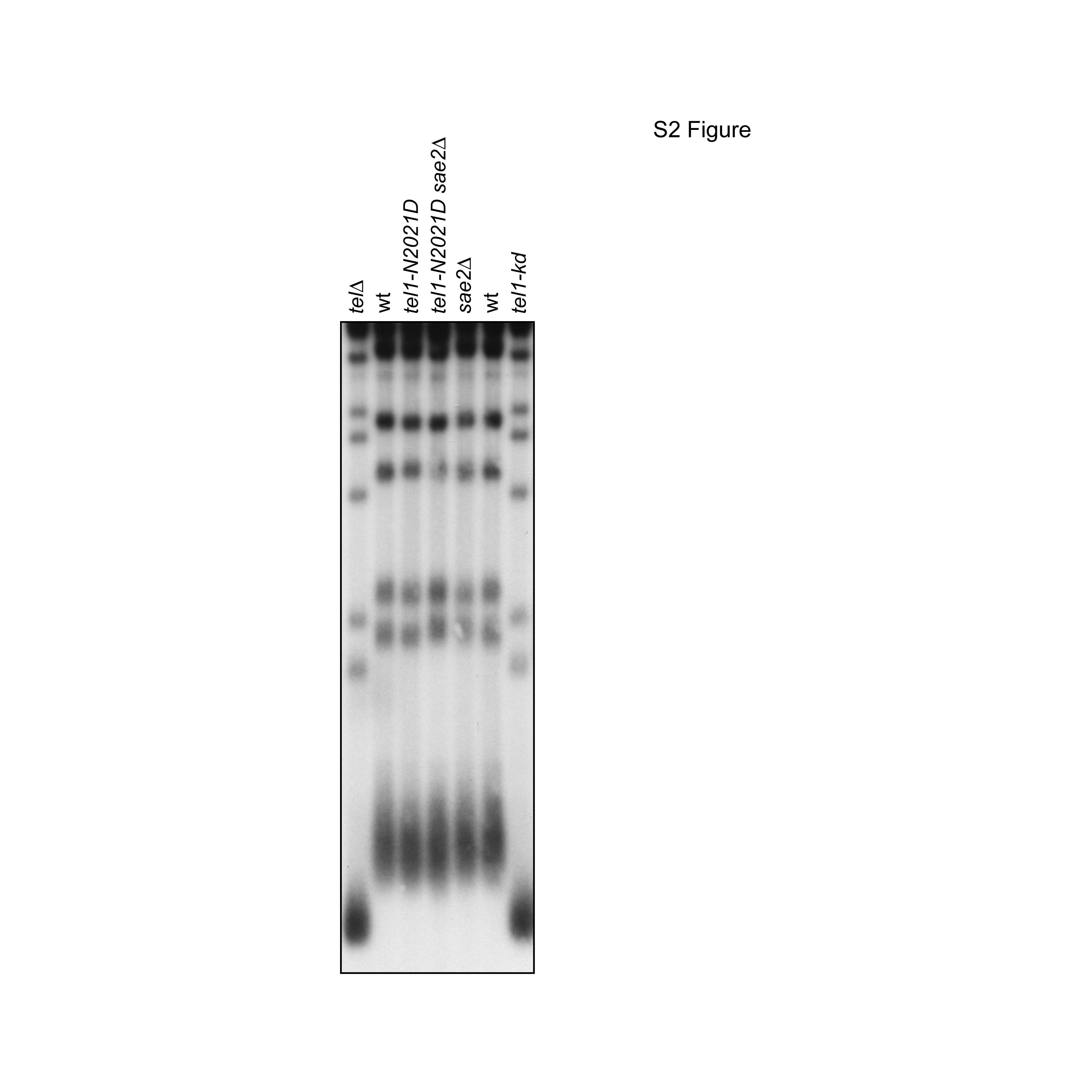

Supplement: S2 Fig — Genomic DNA prepared from exponentially growing cells was digested with XhoI and hybridized with a poly(GT) telomere-specific probe. (TIF) [file pgen.1005685.s002.tif]

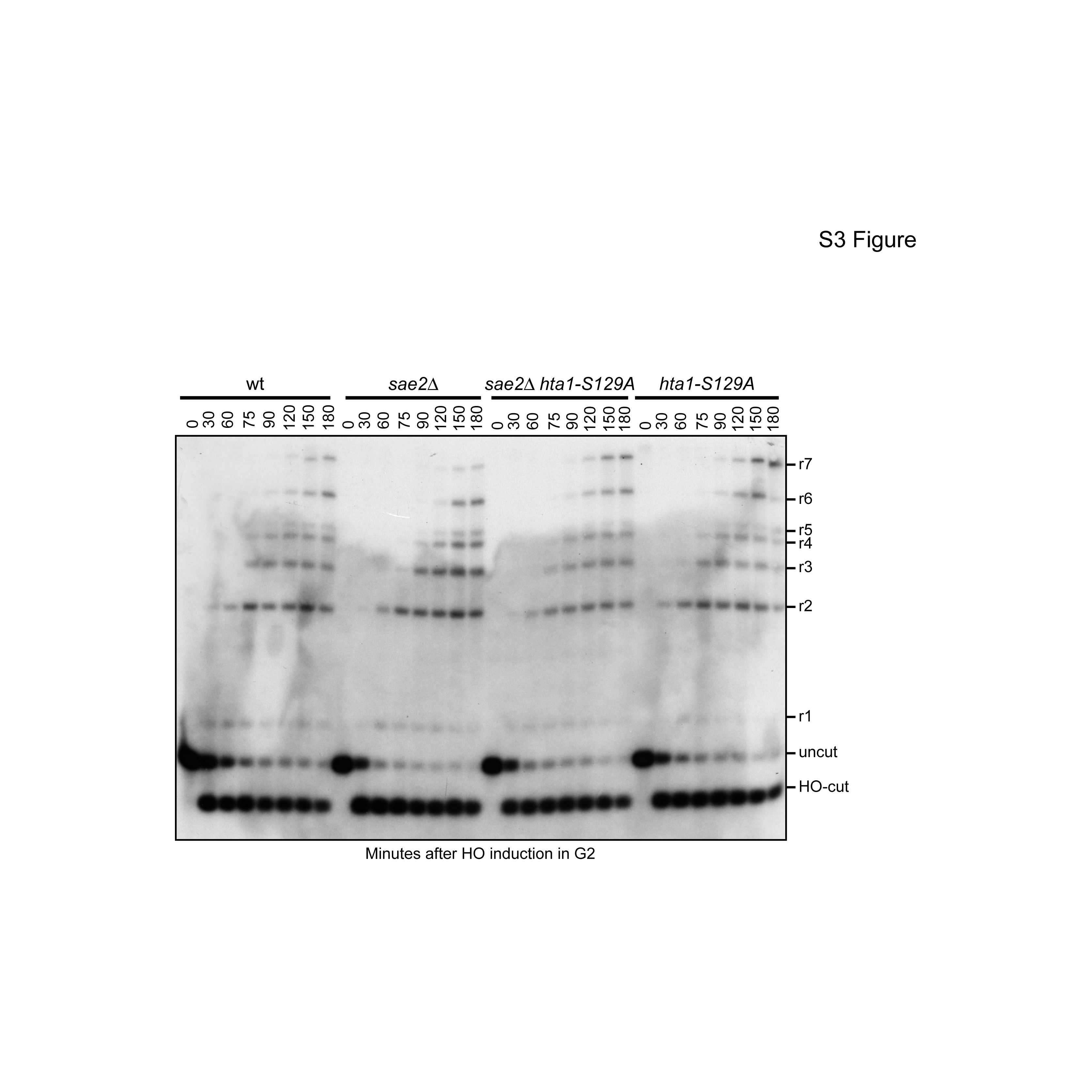

Supplement: S3 Fig — DSB resection. YEPR exponentially growing cultures of JKM139 derivative cells with the indicated genotypes were arrested in G2 with nocodazole and transferred to YEPRG in the presence of nocodazole at time zero. All strains carried also the deletion of HTA2 gene. Gel blots of SspI-digested genomic DNA separated on alkaline agarose gel were hybridized with a single-stranded RNA probe that anneals to the unresected strand on one side of the break. 5’-3’ resection progressively eliminates SspI sites, producing larger SspI fragments (r1 through r7) detected by the probe. (TIF) [file pgen.1005685.s003.tif]
