# Supplementary material for: Sae2 Function at DNA Double-Strand Breaks Is Bypassed by Dampening Tel1 or Rad53 Activity
Source: PLoS Genet. 2015 Nov 19;11(11):e1005685. doi: 10.1371/journal.pgen.1005685 (PMC4652893; doi:10.1371/journal.pgen.1005685)
Supplement: S1 Table — (DOC) [file pgen.1005685.s004.doc]

**Supplementary Information**

**S1 Table.** *Saccharomyces cerevisiae* strains used in this study.

| Strain | Relevant genotype | Source |
| --- | --- | --- |
| W303 | *MAT*a/α *ade2-1 can1-100 his3-11,15 leu2-3,112 trp1-1 ura3-1 rad5-535* | 1 |
| YLL 1069.3 | W303 *sae2∆::KANMX* | This study |
| DMP 6143/8D | W303 *rad53-H88Y::URA3* | This study |
| DMP 5937/1A | W303 *tel1-N2021D::HIS3* | This study |
| DMP 5937/11A | W303 *sae2∆::KANMX tel1-N2021D::HIS3* | This study |
| DMP 5879/3B | W303 *sae2∆::KANMX mre11*Δ*::HIS3* | This study |
| DMP 6283/9D | W303 *sae2∆::KANMX mre11*Δ*::HIS3 rad53-H88Y::URA3* | This study |
| DMP 6272/3A | W303 *sae2∆::KANMX mre11*Δ*::HIS3 tel1-N2021D::HPHMX* | This study |
| DMP 3335/2A | W303 *tel1*Δ*::HIS3* | This study |
| YLL 3575.1 | W303 *tel1-kd*::*LEU2* | This study |
| TEL1-hy909 | W303 *tel1-hy909::LEU2* | 2 |
| DMP 6270/1B | W303 *tel1-hy909::LEU2 sae2∆::KANMX* | This study |
| YLL 3059.1 | W303 *tel1-hy909-kd::KANMX* | This study |
| DMP 6271/3B | W303 *tel1-hy909-kd::KANMX sae2∆::KANMX* | This study |
| DMP 6110/10A | W303 *sae2∆::KANMX dna2-1* | This study |
| DMP 6223/1A | W303 *sae2∆::KANMX dna2-1 rad53-H88Y::URA3* | This study |
| DMP 6110/1C | W303 *sae2∆::KANMX dna2-1 tel1-N2021D::HPHMX* | This study |
| DMP 5058/11B | W303 *sgs1∆::TRP1 exo1∆::HIS3* | This study |
| DMP 6212/5C | W303 *sgs1*∆*::TRP1 exo1∆::HIS3 rad53-H88Y::URA3* | This study |
| DMP 6213/2A | W303 *sgs1∆::TRP1 exo1∆::HIS3 tel1-N2021D::HPHMX* | This study |
| JKM139 | *MAT*a *hml∆*::*ADE1,* *hmr∆::ADE1*, *ade1-100, lys5,*  *leu2-3,112, trp1::hisG ura3-52, ho, ade3::GAL-HO site* | 3 |
| YLL 1523.3 | JKM139 *sae2∆::KANMX* | This study |
| YLL 3622.1 | JKM139 *rad53-H88Y::TRP1* | This study |
| DMP 6013/3A | JKM139 *tel1-N2021D::HPHMX* | This study |
| DMP 6267/3D | JKM139 *rad53-H88Y::URA3 tel1-N2021D::HPHMX* | This study |
| DMP 6267/7D | JKM139 *sae2∆::KANMX rad53-H88Y::URA3 tel1-N2021D::HPHMX* | This study |
| DMP 6074/30B | JKM139 *sae2∆::KANMX rad53-H88Y::URA3* | This study |
| YLL 3532.1 | JKM139 *sae2∆::KANMX tel1-N2021D::HPHMX* | This study |
| YLL 3552.2 | JKM139 *sae2∆::KANMX*  *mre11∆::NATMX::mre11-H125N::URA3* | This study |
| DMP 6217/3A | JKM139 *sae2∆::KANMX mre11-H125N::URA3*  *rad53-H88Y::TRP1* | This study |
| DMP 6041/7D | JKM139 *sae2∆::KANMX mre11-H125N::URA3*  *tel1-N2021D::HPHMX* | This study |
| YLL 1542.1 | JKM139 *sae2∆::KANMX exo1∆::LEU2* | This study |
| DMP 6086/4A | JKM139 *sae2∆::KANMX exo1∆::LEU2 rad53-H88Y::URA3* | This study |
| DMP 6111/8C | JKM139 *sae2∆::KANMX exo1∆::LEU2 tel1-N2021D::HPHMX* | This study |
| YLL 1854.2 | JKM139 *MRE11-18MYC::TRP1* | This study |
| DMP 6149/10A | JKM139 *sae2∆::KANMX MRE11-18MYC::TRP1* | This study |
| DMP 6251/ 5B | JKM139 *rad53-H88Y::URA3 MRE11-18MYC::TRP1* | This study |
| DMP 6149/4A | JKM139 *tel1-N2021D::HPHMX MRE11-18MYC::TRP1* | This study |
| DMP 6251/ 9D | JKM139 *sae2∆::KANMX rad53-H88Y::URA3*  *MRE11-18MYC::TRP1* | This study |
| DMP 6149/11A | JKM139 *sae2∆::KANMX tel1-N2021D::HPHMX*  *MRE11-18MYC::TRP1* | This study |
| DMP 6214/1D | JKM139 *tel1∆::NATMX MRE11-18MYC::TRP1* | This study |
| DMP 6209/10A | JKM139 *tel1-kd::LEU2 MRE11-18MYC::TRP1* | This study |
| YLL 3624.1 | JKM139 *chk1∆::HPHMX* | This study |
| YLL 3625.1 | JKM139 *chk1∆::HPHMX sae2∆::KANMX* | This study |
| 184/10A | JKM139 *mec1∆::HIS3 sml1∆::KANMX* | 4 |
| YLL 3638.1 | JKM139 *rad53-kd::KANMX* | This study |
| DMP 6225/1B | JKM139 *rad53-kd::KANMX sae2∆::KANMX* | This study |
| DMP 6187/3B | JKM139 *tel1-kd::LEU2* | This study |
| DMP 6187/5C | JKM139 *sae2∆::KANMX tel1-kd::LEU2* | This study |
| YLL 2766.7 | JKM139 *sae2∆::HPHMX tel1∆::NATMX* | This study |
| YLL 1794.3 | JKM139 *tel1∆::NATMX* | This study |
| YLL 3222.6 | JKM139 TEL1-3HA::NATMX | This study |
| YLL 3540.1 | JKM139 tel1-N2021D-3HA::NATMX | This study |
| YLL 3670.14 | JKM139 *tel1-kd-3HA::NATMX* | This study |
| DMP 5719/2A | JKM139 *RAD9-3HA::HIS3* | This study |
| DMP 6163/1D | JKM139 *RAD9-3HA::HIS3 rad53-H88Y::URA3* | This study |
| DMP 5816/1A | JKM139 *sae2∆::KANMX RAD9-3HA::HIS3* | This study |
| DMP 6226/5A | JKM139 *rad53-kd::KANMX RAD9-3HA::TRP1* | This study |
| DMP 6208/14A | JKM139 *tel1-kd::LEU2 RAD9-3HA::TRP1* | This study |
| DMP 6226/3C | JKM139 *sae2∆::KANMX rad53-kd::KANMX*  *RAD9-3HA::TRP1* | This study |
| DMP 6208/5D | JKM139 *sae2∆::KANMX tel1-kd::LEU2*  *RAD9-3HA::TRP1* | This study |
| DMP 6023/5A | JKM139 *SGS1-3HA::URA3* | This study |
| DMP 6239/8C | JKM139 *sae2∆::KANMX SGS1-3HA::URA3* | This study |
| DMP 6186/5D | JKM139 *rad53-kd::KANMX SGS1-3HA::URA3* | This study |
| DMP 6240/7A | JKM139 *tel1-kd::LEU2 SGS1-3HA::URA3* | This study |
| DMP 6227/5D | JKM139 *sae2∆::HPHMX rad53-kd::KANMX SGS1-3HA::URA3* | This study |
| DMP 6240/2D | JKM139 *sae2∆::KANMX tel1-kd::LEU2 SGS1-3HA::URA3* | This study |
| YLL 1769.3 | JKM139 *mre11∆::NATMX* | This study |
| YLL 2839. 1 | JKM139 *mre11∆::HPHMX tel1∆::NATMX* | This study |
| DMP 6243/9A | JKM139 *RAD9-3HA::TRP1 hta2∆::NATMX* | This study |
| DMP 6243/18A | JKM139 *RAD9-3HA::TRP1 hta2∆::NATMX sae2∆::KANMX* | This study |
| DMP 6243/5C | JKM139 *RAD9-3HA::TRP1 hta1-S129A::URA3 hta2∆::NATMX sae2∆::KANMX* | This study |
| DMP 5396/4C | JKM 139 *hta2Δ::NATMX* | This study |
| DMP 5129/19B | JKM 139 *hta2Δ::NATMX hta1-S129A::URA3* | This study |
| DMP 5129/34A | JKM 139 *hta2∆::NATMX hta1-S129A::URA3 sae2∆::KANMX* | This study |
| DMP 5129/10B | JKM 139 *hta2∆::NATMX sae2∆::KANMX* | This study |
| YMV45 | *ho hml::ADE1 mata::hisG hmr::ADE1 leu2::leu2(Asp718-SalI)-URA3-*pBR332*-MATa ade3::GAL::HO ade1 lys5 ura3-52 trp1::hisG* | 5 |
| YLL 3646.1 | YMV45 *rad53-H88Y::TRP1* | This study |
| YLL 3548.1 | YMV45 *tel1-N2021D::HPHMX* | This study |
| YLL 1621.9 | YMV45 *sae2∆::KANMX* | This study |
| YLL 3635.2 | YMV45 *sae2∆::HPHMX rad53-H88Y::TRP1* | This study |
| YLL 3533.3 | YMV45 *sae2∆::KANMX tel1-N2021D::HPHMX* | This study |
| YLL 3636.1 | YMV45 *rad53-kd::KANMX* | This study |
| YLL 3628.1 | YMV45 *tel1-kd::HPHMX* | This study |
| YLL 3637.2 | YMV45 *sae2∆:HPHMX* *rad53-kd::KANMX* | This study |
| YLL 3610.1 | YMV45 *sae2∆::KANMX* *tel1-kd::HPHMX* | This study |
| YLL 3529.1 | YMV45 *tel1∆::KANMX* | This study |
| YLL 3563.2 | YMV45 *sae2∆::NATMX tel1∆::KANMX* | This study |

**References**

1. Bonetti D, Martina M, Clerici M, Lucchini G and Longhese MP (2009) Multiple pathways regulate 3' overhang generation at *S. cerevisiae* telomeres. Mol Cell 35: 70-81.
2. Baldo V, Testoni V, Lucchini G, Longhese MP (2008) [Dominant *TEL1-hy* mutations compensate for Mec1 lack of functions in the DNA damage response.](http://www.ncbi.nlm.nih.gov/pubmed/17954565) Mol Cell Biol 28: 358-375.
3. Lee SE, Moore JK, Holmes A, Umezu K, Kolodner RD, Haber JE (1998) *Saccharomyces* Ku70, Mre11/Rad50 and RPA proteins regulate adaptation to G2/M arrest after DNA damage. Cell 94: 399-409.
4. Mantiero D, Clerici M, Lucchini G, Longhese MP (2007) Dual role for *Saccharomyces cerevisiae* Tel1 in the checkpoint response to double-strand breaks. EMBO Rep 8: 380-387.
5. Vaze MB, Pellicioli A, Lee SE, Ira G, Liberi G, Arbel-Eden A, Foiani M, Haber JE (2002) [Recovery from checkpoint-mediated arrest after repair of a double-strand break requires Srs2 helicase.](http://www.ncbi.nlm.nih.gov/pubmed/12191482) Mol Cell 10: 373-385.
